# Multi-Trait Genomic Prediction Improves Predictive Ability for Dry Matter Yield and Water-Soluble Carbohydrates in Perennial Ryegrass

**DOI:** 10.3389/fpls.2020.01197

**Published:** 2020-08-07

**Authors:** Sai Krishna Arojju, Mingshu Cao, Michael Trolove, Brent A. Barrett, Courtney Inch, Colin Eady, Alan Stewart, Marty J. Faville

**Affiliations:** ^1^ Grasslands Research Centre, AgResearch Ltd, Palmerston North, New Zealand; ^2^ Ruakura Research Centre, AgResearch Ltd, Hamilton, New Zealand; ^3^ Barenbrug Agriseeds Ltd, Christchurch, New Zealand; ^4^ Kimihia Research Centre, PGG Wrightson Seeds Ltd, Christchurch, New Zealand

**Keywords:** dry matter yield, genomic selection, nutritive quality, multi-trait genomic prediction, perennial ryegrass

## Abstract

In perennial ryegrass (*Lolium perenne* L), annual and seasonal dry matter yield (DMY) and nutritive quality of herbage are high-priority traits targeted for improvement through selective breeding. Genomic prediction (GP) has proven to be a valuable tool for improving complex traits and may be further enhanced through the use of multi-trait (MT) prediction models. In this study, we evaluated the relative performance of MT prediction models to improve predictive ability for DMY and key nutritive quality traits, using two different training populations (TP1, n = 463 and TP2, n = 517) phenotyped at multiple locations. MT models outperformed single-trait (ST) models by 24% to 59% for DMY and 67% to 105% for nutritive quality traits, such as low, high, and total WSC, when a correlated secondary trait was included in both the training and test set (MT-CV2) or in the test set alone (MT-CV3) (trait-assisted genomic selection). However, when a secondary trait was included in training set and not the test set (MT-CV1), the predictive ability was not statistically significant (p > 0.05) compared to the ST model. We evaluated the impact of training set size when using a MT-CV2 model. Using a highly correlated trait (*r_g_* = 0.88) as the secondary trait in the MT-CV2 model, there was no loss in predictive ability for DMY even when the training set was reduced to 50% of its original size. In contrast, using a weakly correlated secondary trait (*r_g_* = 0.56) in the MT-CV2 model, predictive ability began to decline when the training set size was reduced by only 11% from its original size. Using a ST model, genomic predictive ability in a population unrelated to the training set was poor (*r_p_* = −0.06). However, when using an MT-CV2 model, the predictive ability was positive and high (*r_p_* = 0.76) for the same population. Our results demonstrate the first assessment of MT models in forage species and illustrate the prospects of using MT genomic selection in forages, and other outcrossing plant species, to accelerate genetic gains for complex agronomical traits, such as DMY and nutritive quality characteristics.

## Introduction

Perennial ryegrass is one of the most valued forage species in temperate regions of the world, characterized by relatively high nutritional value, and optimum seasonal and annual dry matter yield (DMY) from herbage, providing a cost effective source of nutrition, for ruminant livestock (Wilkins and Humphreys, 2003; Baert and Muylle, 2016). The historic rate of genetic improvement for seasonal and annual DMY and nutritive quality traits is moderate, between 4% and 7% per decade (Easton et al., 2002; Wilkins and Humphreys, 2003; Sampoux et al., 2011). One reason for the moderate rate genetic improvement is the complex nature of these traits, which makes it challenging to accurately measure. Genomic prediction is considered a valuable tool for improving quantitative traits in both animal and plant breeding (Meuwissen et al., 2001; Habier et al., 2007; Heslot et al., 2015). In genomic prediction, a statistical model is built using genotypic and phenotypic data from a training population and genomic estimated breeding values (GEBVs) are predicted for the non-phenotyped selection candidates using information from genome-wide molecular markers alone (Meuwissen et al., 2001). Compared to traditional breeding approaches, genomic prediction provides opportunities to increase the rate of genetic gain, by reducing the time needed to complete a breeding cycle, increasing the selection intensity and by utilizing within-family variation that can be captured using molecular markers (Heslot et al., 2015; Faville et al., 2018). Indirect selection based on molecular markers (marker assisted selection; MAS) was initially based on quantitative trait loci (QTL) identified in a biparental population or as a subset of significant markers from genome wide association studies (GWAS). MAS has proven to be particularly effective for qualitative traits (Jiang, 2013; Das et al., 2017; Samayoa L et al., 2019), which are controlled by few genes with large effects, whereas, quantitative traits are controlled by many genes with small effects. For quantitative traits, indirect selection based on genomic prediction is considered a more appropriate and practical breeding tool over MAS (Jannink et al., 2010; Heslot et al., 2015). Improvements in next-generation sequencing technology, coupled with the development of reduced representation sequencing approaches such as genotyping-by-sequencing (GBS) (Elshire et al., 2011) for single nucleotide polymorphism (SNP) DNA markers, has made genomic prediction adaptable to forages and other species which lack significant genomics resources such as SNP arrays.

In the context of a genomic selection breeding strategy, higher genetic gains will be achieved for a trait when the prediction model has high predictive ability. Predictive ability is dependent upon trait heritability, training population size, relatedness between training and selection population, statistical model used, marker density, and the extent of linkage disequilibrium (LD) (Daetwyler et al., 2013; De Los Campos et al., 2013). While trait heritability and the extent of LD cannot be easily manipulated, previous studies in perennial ryegrass have developed new knowledge on the effect of relatedness between training and selection population, marker density, and training population size on predictive ability (Fè et al., 2015; Fè et al., 2016; Grinberg et al., 2016; Byrne et al., 2017; Arojju et al., 2018; Faville et al., 2018; Arojju et al., 2020). In perennial ryegrass, genomic prediction models have been developed and validated for many quantitative traits, including DMY (Arojju, 2017; Faville et al., 2018; Guo et al., 2018; Pembleton et al., 2018), nutritive quality traits (Grinberg et al., 2016; Arojju et al., 2020), heading date (Fè et al., 2015; Byrne et al., 2017; Faville et al., 2018; Guo et al., 2018) and crown rust resistance (Fè et al., 2016; Arojju et al., 2018; Guo et al., 2018). In addition, different statistical methods for prediction, with different assumptions regarding the trait inheritance pattern, have been assessed for genomic prediction in this species but little difference in predictive ability was observed (Grinberg et al., 2016; Byrne et al., 2017; Faville et al., 2018).

All of the prediction approaches assessed to date in perennial ryegrass can be categorized as single trait (ST) genomic prediction models, with the majority of them implementing a univariate linear mixed model for predicting GEBVs. In recent years, multi-trait (MT) genomic prediction approaches have been identified as a means of improving predictive ability for a primary trait. In MT genomic prediction, a secondary trait that is genetically correlated with the primary trait is incorporated in the prediction model, to predict the primary trait with higher accuracy (Jia and Jannink, 2012). Multi-trait genomic prediction has been extensively studied in wheat breeding, for example, to improve predictive ability for grain yield using correlated traits, such as canopy temperature (CT) and normalized difference vegetation index (NDVI), measured using high throughput phenotyping (HTP) platforms. Rutkoski et al. (2016) and Sun et al. (2017) reported an improvement of 70% in predictive ability for grain yield when using CT and NDVI as correlated traits in the prediction model. In another study, Sun et al. (2019) reported that a ST genomic prediction model failed to predict grain yield across selection cycles, however, using MT models in which correlated traits CT and NDVI were phenotyped in both the training (cycle 1) and test (cycle 2) sets, the predictive ability for grain yield improved by 146% in the test set. In addition to grain yield, MT models have been validated for improving predictive ability for grain end-use quality traits in wheat. Hayes et al. (2017) used quality trait data acquired from near-infrared spectroscopy (NIRS) and nuclear magnetic resonance (NMR) as correlated traits, to improve end-use quality traits that had been measured using wet-lab assays. Predictive ability was assessed in an independent test set and compared to a ST genomic prediction model. The highest improvement in predictive ability was 21% for loaf volume of bread using NMR predictions as secondary trait in the model.

Training population size can be a limiting factor for implementing genomic prediction for complex traits (Heffner et al., 2009). The optimum training size depends upon the effective population size and the available genetic diversity within the population. For complex traits, such as DMY and nutritive quality, phenotyping a large training population is expensive, which limits the size of training population. Multi-trait models can be used as an approach to increase training population size, by implementing a prediction model wherein the expensive primary trait is phenotyped in a portion of the population, and the less expensive secondary trait is phenotyped in the entire training population. For example, Hayes et al. (2017) predicted end-use quality traits by assembling a large training population, which was partially phenotyped for quality traits measured by wet-lab assay, and completely phenotyped for secondary traits measured using NIR and NMR. In another study, Lado et al. (2018) showed that when a correlated trait was phenotyped in both the training and test set, phenotyping for the primary trait could be reduced to 30% of its original training size, whilst maintaining predictive ability. In MT models, multiple secondary traits may be included for the prediction of a single primary trait. However, there are numerous examples in which inclusion of a single, highly correlated secondary trait in the MT model has proved to be optimal for predicting the primary trait, including biomass in sorghum (Fernandes et al., 2018) and baking quality traits in wheat (Lado et al., 2018). Determining the optimum timing for secondary trait measurement, as well as the number of measurements required, may also increase the impact of MT genomic prediction. For example, in wheat, Sun et al. (2019) reported that predictive ability for grain yield was maximized by scoring the secondary traits (CT and NDVI) approximately 100 to 120 days after planting when in optimal and drought environments but 70 days after planting in hot environments.

For plant species, Jia and Jannink (2012) proposed three statistical models for MT genomic prediction (GBLUP, BayesA and BayesCπ), which have different assumptions regarding the trait inheritance pattern. Based on simulation results, the BayesCπ model performed better for qualitative traits (trait with 20 QTLs), and GBLUP model was more appropriate for quantitative traits (trait with 200 QTLs). Similar results were previously observed for ST genomic prediction models (Daetwyler et al., 2010) suggesting that for both ST and MT genomic prediction, a linear mixed model with additive variance-covariance matrix would be useful for predicting quantitative traits such as DMY and nutritive traits.

In perennial ryegrass, nutritive quality traits measurements using wet-lab assays are preferred, compared to NIR predictions. In this context the MT approach proposed by Hayes et al. (2017), with wet lab measurement of the primary trait and NIRS providing secondary trait data, may be extended to nutritive quality traits in perennial ryegrass, making it feasible to implement genomic prediction for nutritive quality traits in larger training populations. Similarly, visual growth scores are routinely collected in forage breeding programs for biomass estimation. If sufficient genetic correlation exists between visual growth score and DMY, MT genomic prediction approaches may be extended to DMY traits. The objective of the current study was to (i) compare and contrast the relative performance of ST and MT genomic prediction model for DMY and nutritive traits in two different training populations, (ii) evaluate the opportunity to reduce the training population size and predict in an independent population using MT genomic prediction, and (iii) determine the optimum period to phenotype visual growth scores to improve DMY predictive ability.

## Materials and Methods

### Training Population and Experimental Design

Perennial ryegrass training populations, derived from two different breeding programs (Barenbrug Agriseeds Ltd. and Grasslands Innovation Ltd.), were used in this study and designated as TP1 and TP2. Each TP consisted of half-sibling (half-sib) families and maternal parents from five discrete breeding populations (TP1: Pop B1–Pop B5; TP2: Pop I–Pop V).

TP1 was from the Barenbrug Agriseeds Ltd breeding program and was used in the study to investigate MT prediction for DMY. One hundred randomly sampled individuals from each of five F1 generation half-sib families provided by Barenbrug Agriseeds were polycrossed within-family, in separate isolation houses at AgResearch, Palmerston North during spring 2015. F2 generation half-sib seed was harvested from the maternal parents and cleaned prior to sowing field trials during April 2016 at two locations: Ruakura (Waikato region, northern New Zealand, 37.78°S, 175.32°E; Te Rapa peaty silt loam) and Darfield (Canterbury regions, southern New Zealand, 43.45°S, 172.19°E, Hatfield moderately deep silt loam). A total of 463 half-sib families (Pop B1, n = 98; Pop B2, n = 86; Pop B3, n = 98; Pop B4, n = 93; Pop B5, n = 88) were sown at two locations, in a row-column design with three replicates and measured over a period of 3 years. Populations were blocked within each trial, and two repeated checks (n = 103 each) were allocated evenly within and across populations and replicates. The remaining 37 half-sib families yielded insufficient seed for inclusion in the field trials. The unit for evaluation of half-sib families in the trials was a 1-m sown row (plot) of plants (0.3 g seed per plot). Plots were sown with 30 cm spacing between plots. When plants reached two to three leaf growth stages, both trials were defoliated by sheep grazing and if needed, subsequent to grazing, mechanical mowing was applied to residual height of 5 cm. Nitrogen was applied at each defoliation (15 kg N/ha) and superphosphate fertilizer (8.8 kg P/ha) was applied annually in late autumn.

TP2 is the same training population described previously by Faville et al. (2018) and Arojju et al. (2020). This was used in the current study to investigate MT genomic prediction for nutritive traits. Briefly, TP2 was developed by polycrossing 102 to 117 plants from each of five populations in isolation, avoiding admixing between populations, during spring 2012 at AgResearch, Palmerston North, New Zealand. F2 generation half-sib seed from the maternal plants were harvested, cleaned, and subsequently evaluated, a total of 517 families under field conditions. Trials were established in a row-column design with three replicates, during autumn of 2013. Multiple repeated checks were allocated randomly within and across replicates. Within each trial, populations were blocked and within each replicate, families were randomized. For the current experiment, trials at two locations were used to collect data on 18 nutritive quality traits: Trial 1 was located at Lincoln (Canterbury region, southern New Zealand, 43.38°S 172.62°E; Wakanui silt loam) and Trial 2 at Aorangi (Manawatu region, central New Zealand, 40.34°S 175.46°E; Kairanga sandy loam). The unit for evaluation of half-sib families in the trials was a 1-m sown row (plot) of plants (0.2 g seed per plot).

### Phenotypic Data Collection

#### Dry Matter Yield and Growth Score Measurement in TP1

TP1 was phenotyped seasonally for dry matter yield (DMY) and visual growth score (GS) over three years across multiple seasons. The schedule of measurements was as shown in **Supplementary Table 1**, with 10 DMY measurements made at both locations between spring 2016 and summer 2019, and between 18 (Darfield) and 30 (Ruakura) visual GS measurements made during the same period. To assess DMY potential, plots were harvested to a height of 5 cm, and the sample foliage was dried (80°C for 48 h) and weighed to obtain g DM per plot. Harvests were completed when plots were at the two to three leaf growth stage, typically 3 to 4 weeks regrowth following the previous defoliation. The GS measurements were conducted by a single operator at each site on a scale of 0 to 9, with 0 representing no measurable biomass. Where possible, GS data were collected prior to each defoliation event.

#### Nutritive Trait Phenotyping in TP2

From the original set of 18 nutritive quality traits originally assessed in the trial (Arojju et al., 2020) a subset of seven traits (**Supplementary Table 4**) measured from half-sib families were selected for the current study to test the potential of MT genomic prediction approaches. As described in detail by Arojju et al. (2020) half-sib families were harvested on 14^th^ April 2014 at Lincoln and 29^th^ April 2014 at Aorangi. To reduce the influence of diurnal variation on the measured components, harvests were undertaken at each site over three consecutive days, between 10:30 am and 3:00 pm each day. Plots were harvested 5 cm above the pseudostem to collect leaf lamina only and the tissue samples were snap frozen in liquid nitrogen. The collected samples were freeze-dried, milled and split into samples for analysis by AgResearch (Palmerston North, New Zealand) for water-soluble carbohydrate (WSC) using the anthrone reagent assay, and for near-infrared spectroscopy (NIRS) and mineral analysis by Hill Laboratories (Hamilton, New Zealand).

### Genotyping

Genotyping-by-sequencing (GBS) was used to genotype the maternal parents of half-sib families in TP1 and TP2. GBS library preparation, DNA sequencing, data quality assessment, genotype calling and GRM (genetic relationship matrix) estimates largely followed the procedures previously described for TP2 (Faville et al., 2018 and Arojju et al. (2020). The GBS results for TP2 are also detailed in these two publications. Briefly for TP1, we used the same TASSEL-GBS pipeline (Glaubitz et al., 2014) as described for TP2 and obtained 886,597 biallelic SNPs, after removing SNPs with minor allele frequency < 5% and > 50% missing data across all the samples. The 886,597 biallelic SNPs remaining after these filters were exported to KGD software (Dodds et al., 2015). Further filtering based on read depth (> 2) and Hardy-Weinberg disequilibrium (HWdiseq > −0.05) was applied, reducing total SNP number to 547,568. KGD is an approach for GRM estimation which accounts for the (low) read depth of the GBS data and does not require imputation. The KGD generated GRM was used for genomic predictive modelling.

The population structure of TP1 was assessed using multi-dimensional scaling based on the GRM, whilst the population structure used for TP2 was previously reported in Faville et al. (2018).

### Phenotypic Analysis, Heritability, and Correlation

Best linear unbiased predictors (BLUPs) were obtained for the traits measured by analyzing data from across the five breeding populations, for individual locations and across the two locations. In TP1, analysis was performed by fitting a linear mixed model in Genstat (Payne et al., 2002), considering family, family-by-measure, family-by-location interactions, row, column, and replicates as random effects and location, measure, population, and repeated checks as fixed effects in the model. The measure represents the harvest period of DMY and scoring of growth scores in TP1 (**Supplementary Table 1**). Because of similar experimental design, TP2 was analyzed in a manner similar to TP1, but without family-by-measure and measure components in the model. The linear mixed model used to analyze TP2 data was described in detail by Arojju et al. (2020).

For each trait, BLUPs were predicted separately, pooling all five populations across the two locations. The linear mixed model used for generating BLUPs is expressed as:

(1) $$y_{ijklmno}=\mu +g_{i}+s_{m}+{\left(gs\right)}_{im}+{\left(gm\right)}_{in}+p_{o}+b_{onml}+r_{onmlj}+c_{onmlk}+\varepsilon_{ijklmno}$$

*y_ijklmno_* is the vector of phenotypic values measured on half-sib family *i* in row *j* and column *k* of replicate *l* nested in location *m* of measure *n* within population *o*, and *i* = 1,…, *n_g_*, *j* = 1,…, *n_r_*, *k* = 1,…, *n_c_*, *l* = 1,…, *n_b_*, *m* = 1,…, *n_s_*, *n* = 1,…,*n_m_*, *o* = 1,…,*n_p_*, where *g*,*r*,*c*,*b*,*s*,*m*, and *p* are the half-sib families, rows, columns, replicates, locations, measures, and populations, respectively. In the equation, *μ* is the overall mean; *g_i_* is the random effect of half-sib family $i,\;N(0,I\sigma_{g}^{2});\;s_{m}$ is the fixed effect of location *m*; (*gs*)*_im_* is the random effect of interaction between half-sib family *i* and location $m,\;N(0,I\sigma_{gs}^{2});\text{ (}gm{)}_{in}$ is the random effect of interaction between half-sib family *i* and measure $n,\;N(0,I\sigma_{gm}^{2});\;p_{o}$ is the fixed effect of population *o*; *b_onml_* is the random effect of replicate *l* within location *m* of measure *n* in population $o,N(0,I\sigma_{b}^{2});r_{onmlj}$ is the random effect of row *j* within replicate *l* in location *m* of measure *n* in population $o,\;N(0,I\sigma_{r}^{2});\;c_{onmlk}$ is the random effect of column *k* within replicate *l* in location *m* of measure *n* in population $o,\;N(0,I\sigma_{c}^{2});\;\varepsilon_{ijklmno}$ is the residual effect of half-sib family *i* in row *r* and column *c* of replicate *b* in location *m* of measure *n* in population $o,\;N(0,I\sigma_{\varepsilon }^{2})$. For individual locations, BLUPs were estimated using a mixed model equivalent to Eq. 1, without (*gs*)*_im_* and *s_m_* component in the model.

Genomic heritability (*h^2^_g_*) was estimated using Eq. 1, fitting variance-covariance structure among genotypes as follows, $g∼N(0,G\sigma_{g}^{2})$, where *G* is the KGD genomic relationship matrix. The mixed model was fitted using ASReml-R (Butler et al., 2009) to estimate variance components. The additive genetic variance was estimated as the proportion of variance explained by regressing markers on phenotypes (De Los Campos et al., 2015) and *h^2^_g_* was calculated as follows:

(2) $$h_{g}^{2}=\frac{\sigma_{g}^{2}}{\sigma_{g}^{2}+\frac{\sigma_{gs}^{2}}{s}+\frac{\sigma_{gm}^{2}}{m}+\frac{\sigma_{\varepsilon }^{2}}{smb}}$$

where, $h_{g}^{2}$ is the genomic heritability, $\sigma_{g}^{2}$ is the additive genetic variation among half-sib families, $\sigma_{gs}^{2}$ is the variance associated with family-by-location interaction, $\sigma_{gm}^{2}$ is the variance associated with family-by-measure interaction and $\sigma_{\varepsilon }^{2}$ is the residual variance.

The Pearson correlation coefficient between primary trait and secondary trait was estimated using BLUPs generated from Eq. 1 in R statistical programming language (R Core Team, 2017) as follows:

(3) $$r_{g(x,y)}=\frac{Cov_{g(x,y)}}{\sqrt{\sigma^{2}(x),\sigma^{2}(y)}}$$

where, *r_g_*_(_ *_x,y_*_)_ is the correlation between secondary and primary trait, *Cov_g_*_(_ *_x,y_*_)_ is the covariance between trait *x* and *y*; *σ* ^2^(*x*) is the variance associated with trait *x*, and *σ* ^2^(*y*) is the variance associated with trait *y*.

### Genomic Prediction Model and Cross-Validations

Genomic estimated breeding values (GEBVs) were predicted using BLUPs from Eq. 1 as the dependent variable in the linear mixed model. The Reproducing Kernel Hilbert Space (RKHS) was used as a single trait (ST) genomic prediction model, which is equivalent to the Genomic BLUP (GBLUP) model when kernel is linear (Cuevas et al., 2017). The RKHS was implemented using a Bayesian framework by considering the reproducing kernel (RK) as a marker derived additive relationship matrix (RK = KGD matrix) (Pérez and De Los Campos, 2014).

For both TP1 and TP2, a linear mixed model was fitted as follows:

(4) y=β+Zα+ε

where *y* is the BLUP values of the trait from the mixed model, *β* is the vector of grand mean, *Z* is the design matrix associated with random marker effects *α*, RKHS assumes that marker effects are distributed as $\alpha ∼N(0,\;K\sigma_{\mu }^{2})$, where *K* is an (n x n) additive genetic relationship matrix, and random residual errors as $\varepsilon ∼N(0,I\sigma_{\varepsilon }^{2})$, in which *I* is the identity matrix. The additive relationship matrix was calculated using KGD method (Dodds et al., 2015). A single kernel model was implemented with 3,000 iterations and 1,500 burn-in using BGLR package in R (Pérez and De Los Campos, 2014).

For multi-trait (MT) genomic prediction, a bivariate linear mixed model was fitted using two correlated traits as follows:

(5) $$\left[\begin{array}{c} y_{1}\\ y_{2} \end{array}\right]=\left[\begin{array}{c} \beta_{1}\\ \beta_{2} \end{array}\right]+\left[\begin{array}{cc} Z_{1} & 0\\ 0 & Z_{2} \end{array}\right]\left[\begin{array}{c} \alpha_{1}\\ \alpha_{2} \end{array}\right]+\left[\begin{array}{c} \varepsilon_{1}\\ \varepsilon_{2} \end{array}\right]$$

where *y*_1_ and *y*_2_ are the vector of BLUP values for trait 1 and trait 2, *β*_1_ and *β*_2_ are the vector grand means, *Z*_1_ and *Z*_2_ are the design matrices associated with random effects *α*_1_ and *α*_2_, in which ${(}_{\alpha_{2}}^{\alpha_{1}})∼N(0,G⊗K),$ where *K* is the marker-based relationship matrix (KGD matrix) and *G* is the genetic variance-covariance matrix for all traits, *ε*_1_ and *ε*_2_ are the random residuals from bivariate models, with ${(}_{\varepsilon_{2}}^{\varepsilon_{1}})∼N(0,I⊗R),$ where *I* is the identity matrix, and *R* is the residual variance-covariance matrix between two traits. The model was implemented assuming *K* as unstructured matrix and *R* as diagonal matrix (Lado et al., 2018), with 3,000 iterations and 1,500 burn-in using the MTM R package (De Los Campos and Grüneberg, 2016).

**Figure 1:**
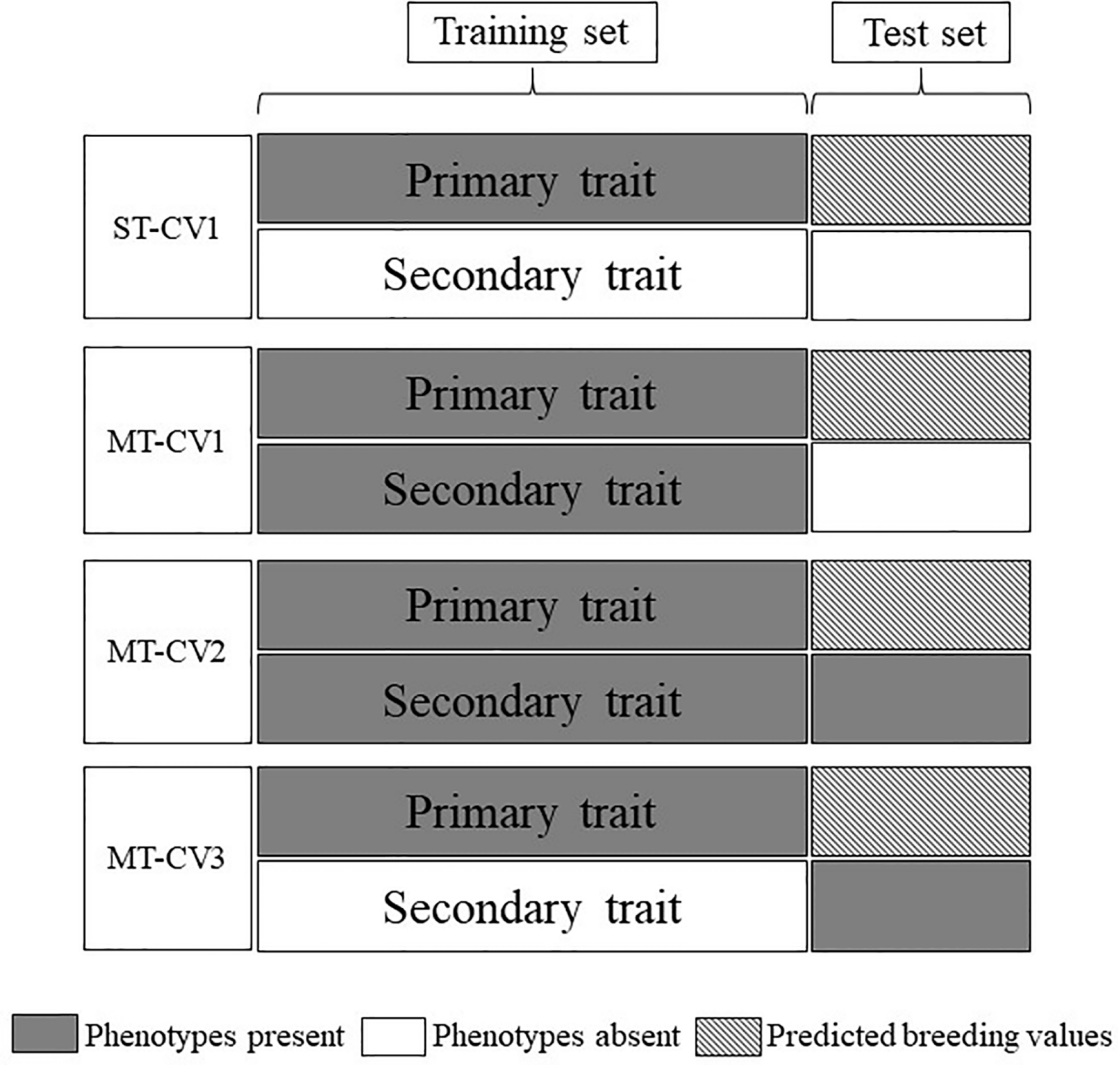
Cross-validation schemes employed for single-trait (ST) and multi-trait (MT) genomic prediction models. Data were divided into training and test sets. Gray boxes indicate phenotypes were present in the model and white boxes indicate the absence of phenotypes. Hatched boxes indicate the primary trait was predicted in the test set using either ST or MT models.

The predictive ability of ST and MT genomic prediction models was assessed using a Monte-Carlo cross-validation (CV) approach. For MT models, three different CV strategies were performed, CV1 CV2 and CV3 (**Figure 1**).

The CV1 approach was applied to both ST and MT models (ST-CV1 and MT-CV1, respectively). A random 70% of the TP genotypic and phenotypic data set (primary + secondary traits for MT-CV1) was used as a training set to train the genomic prediction model and the remaining 30% set aside as the test set. GEBVs were predicted for the test set individuals, and this process was iterated 100 times. The predictive ability of the model was the mean Pearson correlation coefficient between GEBVs and the observed phenotypes in the test set.

The CV2 and CV3 cross-validation approaches were assessed for the MT model only (MT-CV2, MT-CV3, respectively). For MT-CV2, the methodology was consistent with previous studies (Jia and Jannink (2012); Sun et al. (2017); Fernandes et al. (2018); Sun et al. (2019). A random 70% of genotypic and primary trait phenotypic data (training set) were assigned to train the model, along with 100% of the phenotypic data from the secondary (correlated) trait. In other words, in contrast to MT-CV1, in the MT-CV2 scenario the 30% of individuals comprising the test set also had phenotypic data for a secondary trait. The GEBVs for the primary trait were predicted for the individuals in the test set. This process was iterated 100 times, and the predictive ability of the model was the mean Pearson correlation coefficient between GEBVs and the observed phenotypes. The MT-CV3 approach was implemented similarly to MT-CV2, with the exception that in this scenario phenotypes for the secondary trait were present only in the test set and were excluded from the training set (**Figure 1**).

### Predicting in an Independent Population

Training populations TP1 and TP2 were both composites of five breeding populations each. To test the feasibility of genomic prediction in a population that is genetically independent of the training set, genomic prediction models were trained using four breeding populations and used to predict GEBVs in the fifth. Predictive ability was assessed for the trait DMY All Cuts in TP1 and for the trait Total WSC in TP2, using the ST-CV1 and MT-CV2 approaches. A further evaluation was applied with the MT-CV2 approach, by testing using a single secondary trait with either low or high correlation to the primary trait. This procedure was repeated five times, with a different breeding population used as the test set each time.

### Optimizing Training Population Size

A random set of half-sib families, ranging from 10% to 90% of the full training population (TP1, n = 36 to 329 and TP2, n = 41 to 369), were selected as a training set to evaluate the impact of training set size on predictive ability. In both TP1 and TP2, the predictive ability for a trait for each of the different-sized training sets was assessed in a test set made up of 20% of training population individuals, using both ST-CV1 and MT-CV2 approaches. As mentioned above, the MT-CV2 approach was further investigated by using a single secondary trait in the prediction model, either low or high correlation to the primary trait, for both TP1 and TP2.

## Results

### Genomic Heritability, Correlation, and Population Structure

The observed family variance components for all DMY and GS traits measured in TP1 were significant (P < 0.05) (**Table 1**). Family-by-location and family-by-measure interactions were also significant (P < 0.05), expect for DMY Late Spring and GS Late Spring (**Table 1**). For DMY traits, the family variance components were large compared to variance components associated with family-by-location. However, for GS traits, variance components for family-by-location interactions exceeded family variance (**Table 1**). For both DMY and GS traits, family-by-measure interaction variance components were comparatively small (**Table 1**). The family variance components measured in the Ruakura trial were larger than Darfield, and this was reflected in the genomic heritability for DMY and GS traits, which were consistently higher at Ruakura (**Supplementary Tables 2** and **3**). The genomic heritability for DMY traits phenotyped in TP1 were medium to high, from 0.35 to 0.65, based on mean performance across the two locations, Ruakura and Darfield (**Table 1**). The highest genomic heritability was 0.65 for DMY All Cuts (average DM across all measures) and the lowest genomic heritability was 0.35 for DMY Spring. For GS traits, the genomic heritability was low compared to DMY traits, ranging from 0.21 to 0.46 (**Table 1**). The GS Summer was highly heritable (0.46) and the lowest genomic heritability was observed for GS Spring (0.21). The correlations between DMY and GS traits were highly positive (**Table 2**). DMY All Cuts was positively correlated with all GS traits, the highest correlation being *r_g_* = 0.88 with GS All (average GS across all measures) and the lowest correlation was with GS Spring (*r_g_* = 0.56). Among seasonal DMY traits, DMY Autumn was the most highly correlated with all GS traits (mean *r_g_* = 0.70), followed by DMY summer (mean *r_g_* = 0.66), DMY Spring (mean *r_g_* = 0.63) and DMY Late Spring (mean *r_g_* = 0.62) (**Table 2**).

**Table 1:** Training population (TP) 1 was measured for seasonal and mean dry matter yield (DMY) and visual growth scores (GS) across two locations and 3 years.

Trait description	Trait	σ^2^_g_ ± SE	σ^2^_gs_ ± SE	σ^2^_gm_ ± SE	*h^2^_g_*
Dry matter yield	DMY All Cuts	10.09 ± 1.412	6.74 ± 0.691	1.18 ± 0.295	0.65
	DMY Spring	4.89 ± 1.048	5.12 ± 1.016	1.44 ± 0.519	0.35
	DMY Late Spring	11.66 ± 2.050	4.60 ± 1.391	1.23 ± 0.938^†^	0.50
	DMY Summer	15.06 ± 2.254	8.41 ± 1.153	2.15 ± 0.793	0.61
	DMY Autumn	11.41 ± 1.836	2.05 ± 0.989	0.00 ± 0.000	0.52
Growth score	GS All	0.08 ± 0.014	0.29 ± 0.015	0.07 ± 0.004	0.35
	GS Spring	0.03 ± 0.009	0.16 ± 0.013	0.05 ± 0.010	0.21
	GS Late Spring	0.09 ± 0.018	0.16 ± 0.018	0.02 ± 0.012^†^	0.34
	GS Summer	0.14 ± 0.022	0.27 ± 0.017	0.04 ± 0.007	0.46
	GS Autumn	0.14 ± 0.026	0.49 ± 0.030	0.01 ± 0.004	0.34
	GS Winter	0.09 ± 0.018	0.20 ± 0.017	0.09 ± 0.013	0.38

^†^non-significant at P > 0.05.

Shown are family genetic variance (σ^2^_g_), family-by-site interactions (σ^2^_gs_), family-by-measure interactions (σ^2^_gm_), and associated standard errors (SE), and genomic heritability (h^2^_g_) among perennial ryegrass half-sib families across the two locations.

**Table 2:** Pearson correlation coefficient between dry matter yield (DMY) traits and visual growth score (GS) traits, measured among perennial ryegrass half-sib families in training population TP1, at two locations.

Trait	GS Spring	GS Late Spring	GS Summer	GS Autumn	GS Winter	GS All
DMY All Cuts	0.56	0.61	0.81	0.86	0.71	0.88
DMY Spring	0.65	0.56	0.57	0.60	0.72	0.67
DMY Late Spring	0.55	0.62	0.62	0.64	0.60	0.68
DMY Summer	0.44	0.52	0.77	0.83	0.60	0.8
DMY Autumn	0.46	0.56	0.80	0.85	0.67	0.83

DMY All Cuts, mean across all DMY harvests; GS All, mean GS across all seasonal GS.

Genomic heritability and variance components for nutritive traits phenotyped in TP2 were previously reported by Arojju et al. (2020). Genomic heritability for the seven nutritive traits included in the current study were low to high (0.21 to 0.48) and the family variance was significant for all the seven traits (**Supplementary Table 4**). Family-by-location interactions were significantly higher for WSC traits compared to ADF, NDF, and DOMD. Strong positive correlations were observed between DOMD and WSC traits and a strong negative correlation occurred between NDF and WSC traits (Arojju et al., 2020).

**Figure 2:**
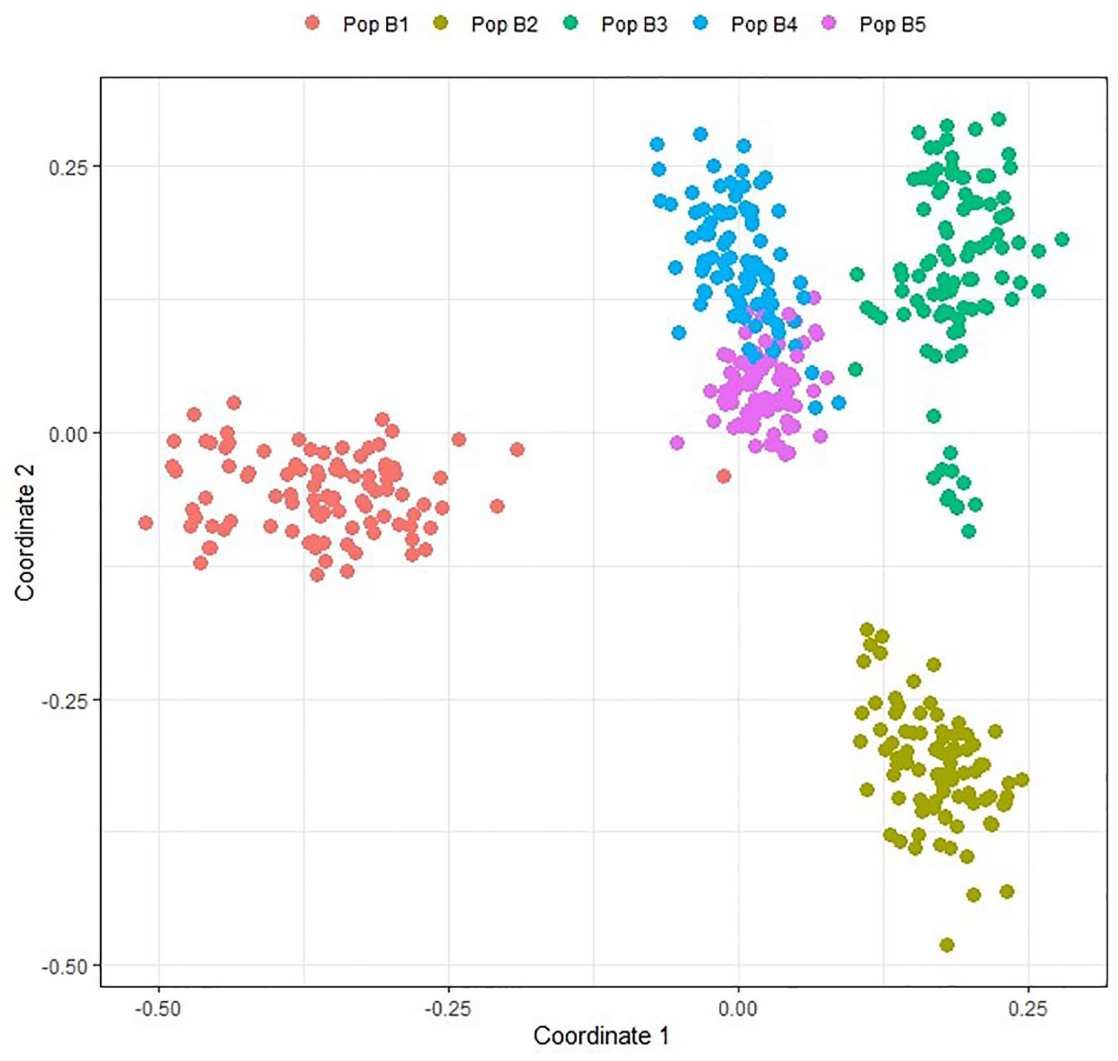
Multidimensional scaling plot of individuals in training population TP1 based on data from 547,568 GBS SNP markers. TP1 is composed of individuals from five discrete populations, Pop B1, Pop B5.

Population structure in the TP1 training set was estimated using multi-dimensional scaling (MDS) based on the GRM. MDS analysis revealed clustering of individuals into five groups, corresponding to the five breeding populations making up the training set (**Figure 2**). Two populations (B4 and B5) shared common individuals, and the grouping was closer compared to other populations due to shared lineage.

### Single-Trait Genomic Prediction

**Figure 3:**
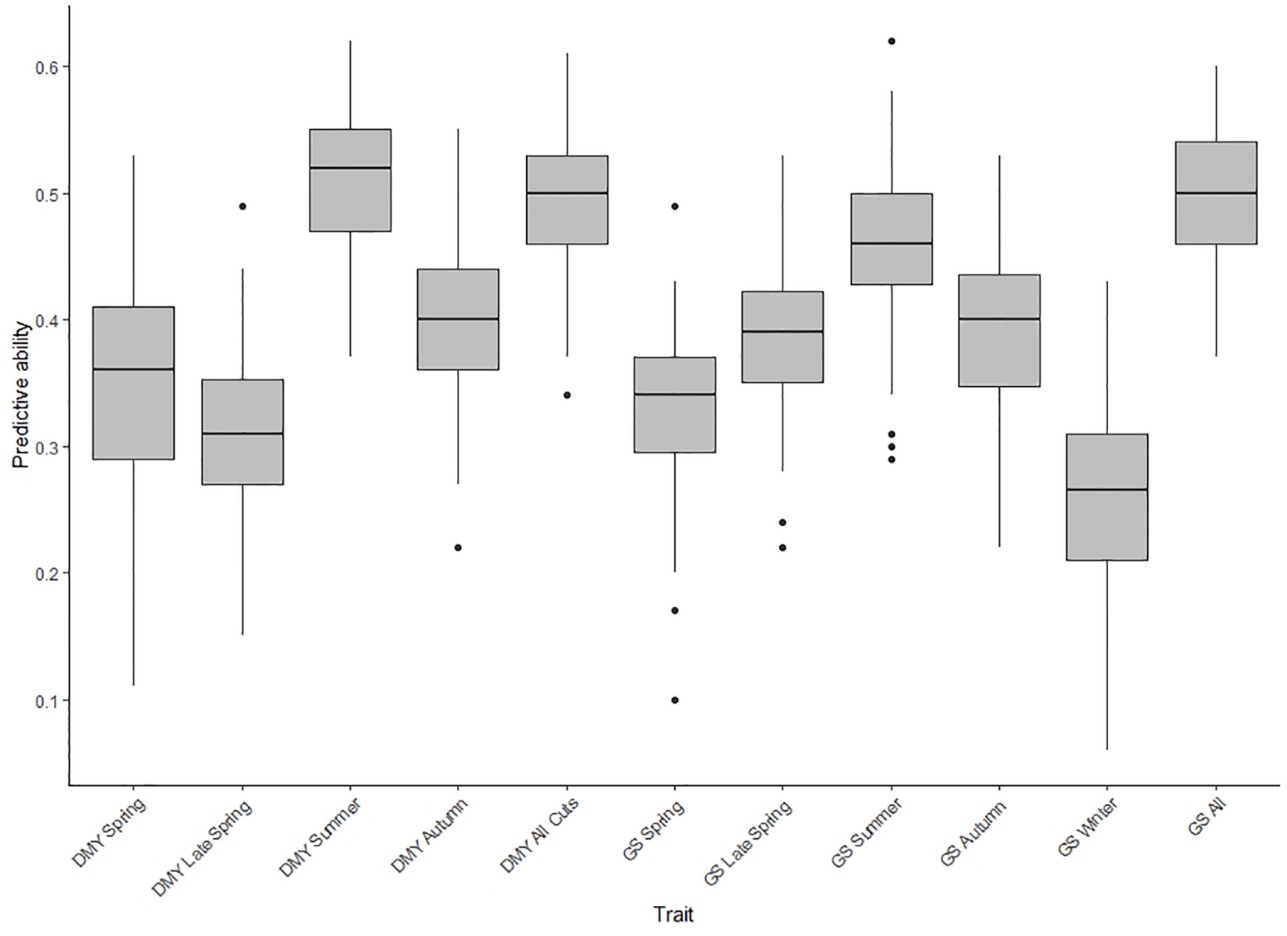
Box plot of predictive abilities for seasonal and combined (All Cuts) dry matter yield (DMY) traits and seasonal and combined (All Cuts) growth scores (GS) in training population TP1, assessed using a single trait genomic prediction model (ST-CV1 approach) based on across-location best linear un-biased predictors (BLUPs).

Based on BLUPs estimated across the two locations, the predictive ability for DMY All Cuts and GS All in TP1, was 0.50 for both, with an unbiased estimate (**Figure 3** and **Supplementary Figure 1**). The predictive ability for across-location seasonal DMY and GS traits were from 0.26 to 0.51 (bias ~ 1; slope of regression) (**Figure 3** and **Supplementary Figure 1**). The predictive abilities for Spring, Summer and Autumn DMY were higher compared to the equivalent seasonal GS traits. The seasonal trend for predictive ability was similar when comparing DMY and GS traits with the exception of the late spring values (**Figure 3**). Among 11 traits assessed for predictive ability, the highest was observed for DMY Summer (*r_p_* = 0.51) and low predictive ability was observed for GS winter (*r_p_* = 0.26). Based on BLUPs estimated only from Ruakura, the predictive ability for DMY and GS traits ranged from 0.25 to 0.62 (**Supplementary Table 2**). By contrast, using BLUPs from Darfield only the predictive ability was relatively low for DMY and GS traits (*r_p_* = 0.20 to 0.40) (**Supplementary Table 3**). In Ruakura, the predictive ability for GS All was 0.62 and the predictive ability for DMY All Cuts was 0.46 (**Supplementary Table 2**) overall, predictive ability was higher for GS traits compared to DMY traits (**Supplementary Table 2**). In Darfield, the predictive ability was similar for DMY and GS traits and was low compared to Ruakura (**Supplementary Table 3**).

**Figure 4:**
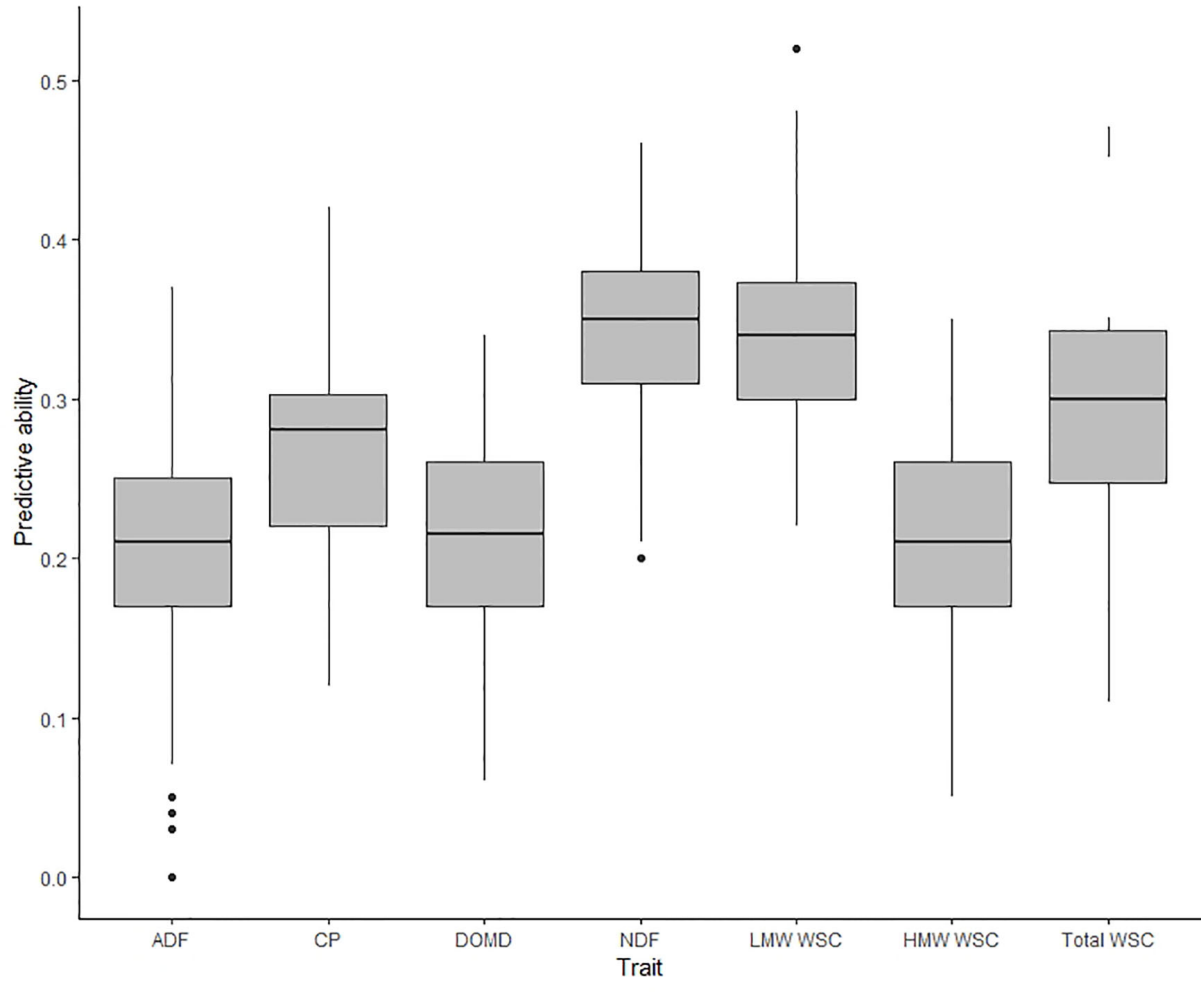
Box plot of predictive abilities estimated for nutritive traits measured in training population TP2 (Arojju et al., 2020) using a single trait (ST-CV1) genomic prediction model.

The predictive ability for nutritive traits estimated in TP2 were previously reported by Arojju et al. (2020) using GBLUP, KGD-GBLUP, and BayesCπ genomic prediction models. In the current study, predictive ability was estimated using RKHS, wherein the statistical assumptions were equivalent to the GBLUP and KGD-GBLUP models (Cuevas et al., 2017). Predictive ability for the seven traits using RKHS was within the range of previously reported values (see **Figure 1**, Arojju et al. (2020)) expect for ADF which decreased by 20% in predictive ability using the RKHS model (**Figure 4**). For all seven nutritive traits, predictive ability estimated using RKHS model was moderate, from 0.20 to 0.35, with an unbiased estimate (**Figure 4** and **Supplementary Figure 2**). The highest predictive ability among seven nutritive traits was observed for NDF (*r_p_* = 0.35) and the lowest predictive ability was for ADF (*r_p_* = 0.20). When the model was based on BLUPs estimated from Aorangi, the predictive ability was higher for all seven nutritive traits in comparison to Lincoln (**Supplementary Table 4**). The predictive ability using BLUPs from Lincoln, was consistently low (NDF, CP, HMW WSC, and Total WSC) and in some cases negative (ADF, DOMD, and LMW WSC) (**Supplementary Table 4**).

### Multi-Trait Genomic Prediction

**Figure 5:**
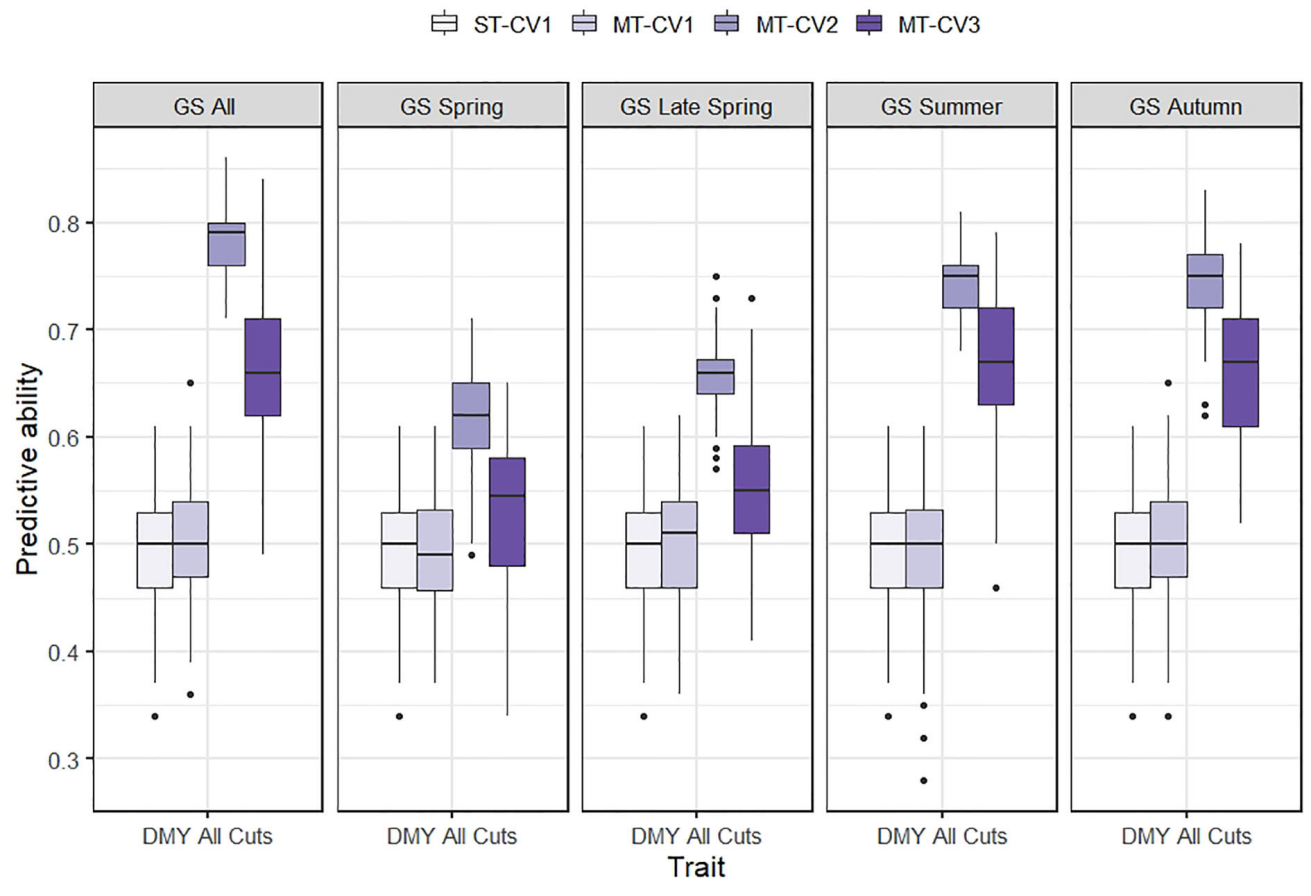
Box plot of predictive ability for mean dry matter yield from all seasonal harvests (DMY All Cuts) in TP1 using single-trait (ST-CV1) and multi-trait (MT-CV1, MT-CV2, and MT-CV3) genomic prediction models. In ST-CV1 model, DMY All Cuts was used to train and predict without any secondary trait in the model. In MT-CV1, DMY All Cuts and one of five correlated secondary traits (GS All, GS Spring, GS Late Spring, GS Summer and GS autumn) were used to train the model and predict DMY All Cuts in the test set. Under the MT-CV2 scheme, phenotypic data of a secondary trait was used in both the training and test set to predict DMY All Cuts. The MT-CV3 approach is similar to MT-CV2, except that phenotypes of the secondary trait were excluded from the training set, leaving secondary trait phenotypes in the test set only, for prediction of the primary trait.

**Figure 6:**
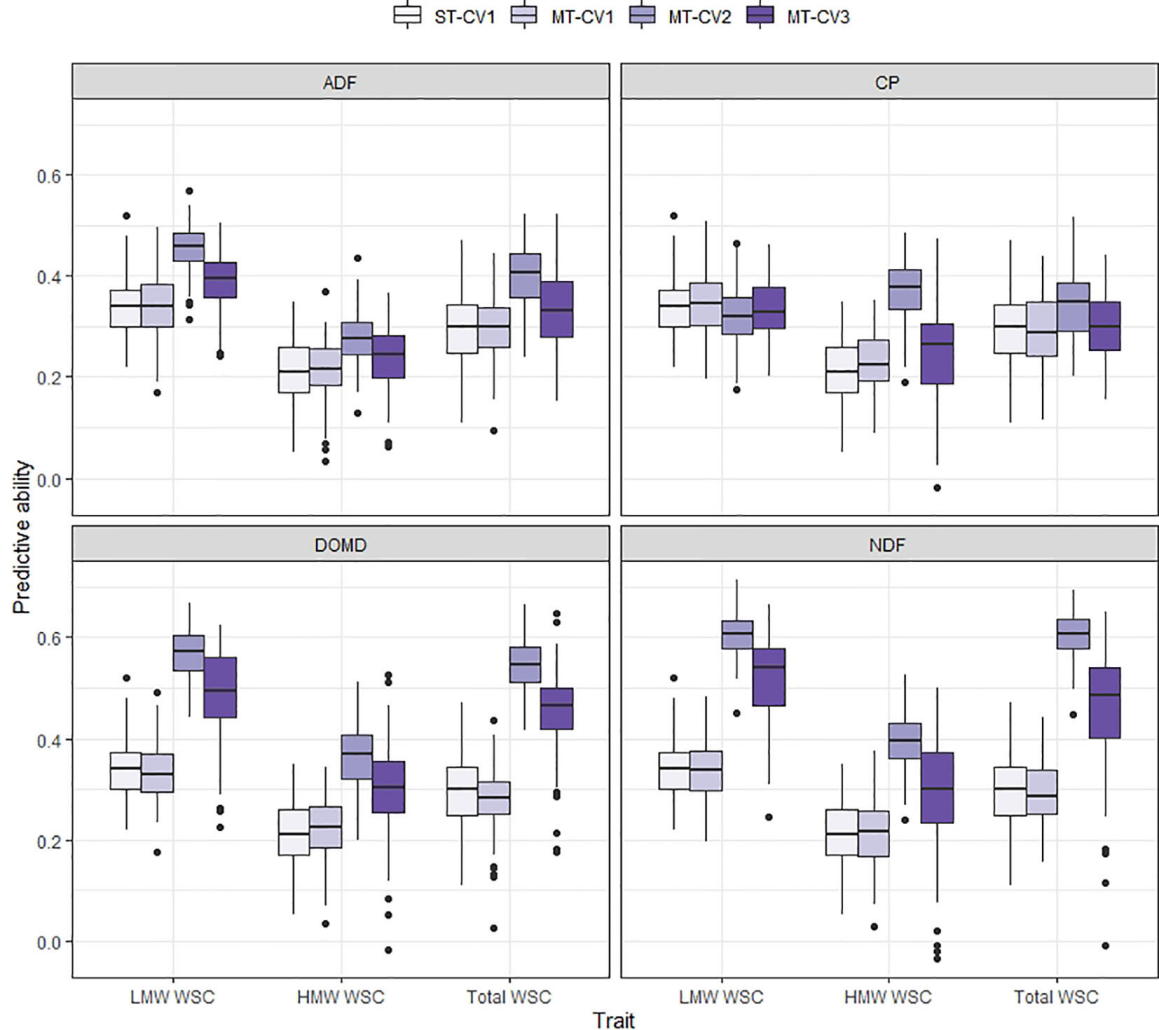
Box plot of predictive ability for LMW WSC, HMW WSC, and Total WSC traits assessed in TP2 using single-trait (ST-CV1) and multi-trait (MT-CV1, MT-CV2, and MT-CV3) genomic prediction models. In ST-CV1, the primary trait (LMW WSC, HMW WSC, and Total WSC) was used to train and predict without any secondary trait in the model. In MT-CV1, primary and secondary trait data (one of ADF, NDF, DOMD, or CP) were used to train the model and predict the primary trait in test set. Under the MT-CV2 scheme, phenotypic data of the secondary trait was used in both the training and test set to predict the primary trait. The MT-CV3 approach is similar to MT-CV2, except that phenotypes of the secondary trait were excluded from the training set, leaving secondary trait phenotypes in the test set only, for prediction of the primary trait.

Using the MT-CV1 genomic prediction scheme (individuals in the training set phenotyped for primary and secondary trait; individuals in test set with no phenotypic information), the predictive ability for DMY All Cuts in TP1 and for WSC traits in TP2 was similar to that of the ST genomic prediction models (ST-CV1) (**Figures 5** and **6**). However, under MT-CV2, wherein individuals in the test set had phenotypic information for a secondary (correlated) trait, predictive ability for DMY All Cuts (TP1) and WSC traits (TP2) was significantly improved (**Figures 5** and **6**). Under the MT-CV3 scheme, wherein individuals in the training set had phenotypic information for the primary trait only but those in the test set were phenotyped for a secondary trait (**Figure 1**, MT-CV3), there was an improvement in predictive ability for both DMY All Cuts and WSC traits, compared to ST-CV1 (**Figures 5** and **6**). However, for both DMY All Cuts (TP1) and WSC traits (TP2), the MT-CV2 model always generated the highest predictive abilities compared to the ST-CV1 and MT-CV3 approaches (**Figures 5** and **6**).

In TP1, the use of GS traits as secondary traits in the MT-CV2 model improved the predictive ability for DMY All Cuts by 24% to 59% compared to ST-CV1 (**Figure 5**). Enhancement of predictive ability under MT-CV2 was dependent upon the size of the correlation between secondary and primary traits. Using GS All as the secondary trait, which had the highest correlation with DMY All cuts (*r_g_* = 0.88) the predictive ability increased by 59%. The lowest correlation was observed between GS Spring and DMY All cuts (*r_g_* = 0.56) and the improvement in predictive ability in this model was the smallest (25%). For GS Summer and GS Autumn, the improvement in predictive ability was similar (51%), although the correlation with DMY All Cuts was slightly higher for GS Autumn (*r_g_* = 0.86), compared to GS Summer (*r_g_* = 0.81). In the MT-CV3 model, using GS All as a secondary trait, improved predictive ability for DMY All Cuts by 34% compared to ST-CV1 (**Figure 5**). Using seasonal GS traits to predict DMY All Cuts, the improvement in predictive ability ranged from 8% to 36% under the MT-CV3 approach (**Figure 5** and **Supplementary Figure 3**).

In TP2, the highest improvement in predictive ability was observed when including secondary traits DOMD or NDF in the MT-CV2 model for the prediction of LMW, HMW or Total WSC as the primary traits (predictive ability improved by 67% to 105% compared to ST-CV1 model) (**Figure 6**). For Total WSC, the highest improvement in predictive ability was 105%, when using NDF as a secondary trait, and the lowest improvement was 16% with CP as the secondary trait. There was a decrease in predictive ability for MT-CV2, compared to ST-CV1, by 5% for LMW WSC, when CP was used as a secondary trait. This was due to the absence of correlation between the two traits (*r_g_* = −0.05; Arojju et al. (2020)). Under the MT-CV3 scheme, similar to MT-CV2, using DOMD and NDF as secondary trait gave the highest improvements in predictive ability for LMW, HMW, and Total WSC traits (39% to 59% improvement in predictive ability compared to ST-CV1) (**Figure 6** and **Supplementary Figure 4**).

### Predicting in an Independent Population

**Figure 7:**
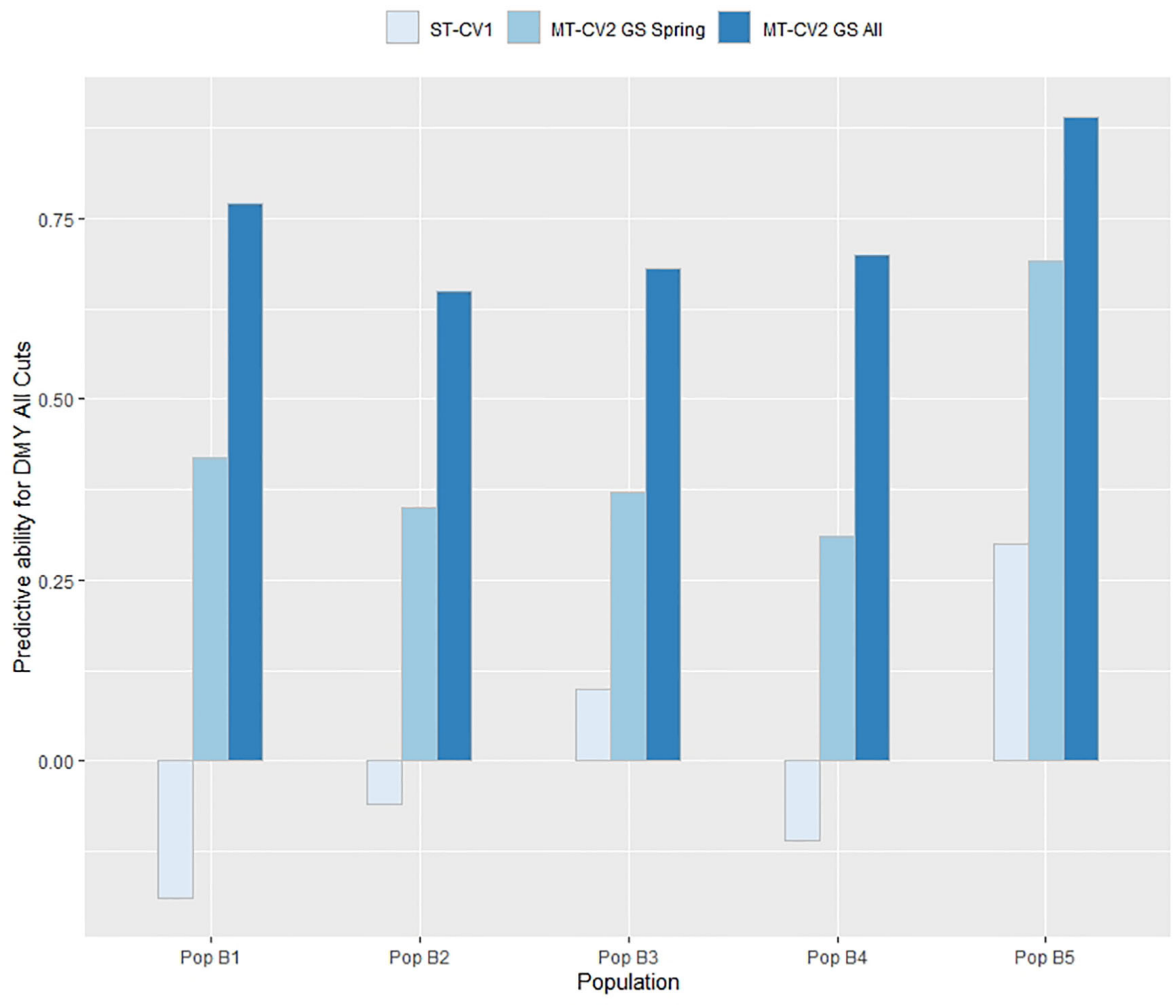
Predictive ability for DMY All Cuts in each population within TP1 was estimated for genomic prediction models using genotypic and phenotypic data from four populations as a training set and the remaining fifth population as a test set. This was repeated five times with a different population as test set each time. Predictive ability was estimated using single trait (ST-CV1) and multi-trait (MT-CV2) genomic prediction models, with the MT-CV2 model further assessed by comparing use of a low correlated secondary trait (GS Spring) and a high correlated secondary trait (GS All Cuts).

The efficacy of genomic prediction in an independent population was assessed by using individuals from four of the breeding populations in the overall training set (TP1 or TP2) to train a model that was subsequently used to predict individuals in the fifth population. In TP1, using the ST-CV1 approach the predictive ability for DMY All Cuts was only positive when predicting individuals in Pop B3 and Pop B5 (*r_p_* = 0.1 and 0.3, respectively) whereas the predictive ability in Pop B1, B2, and B3 was negative (*r_p_* = −0.06 to −0.19) (**Figure 7**). Using the MT-CV2 approach, with a low correlated secondary trait (GS spring; *r_g_* = 0.56) in the model, the predictive ability for DMY All Cuts was positive when predicting in all five populations, with values ranging from 0.31 to 0.69 (**Figure 7**). Predictive ability for DMY All Cuts in each population, when using a highly correlated secondary trait (GS All; *r_g_* = 0.88) was highest, ranging from 0.65 to 0.89 (**Figure 7**). Using a highly correlated secondary trait (GS All) in the MT-CV2 model predictive ability improved by 125% above the MT-CV2 models constrained to using a low correlated secondary trait.

**Figure 8:**
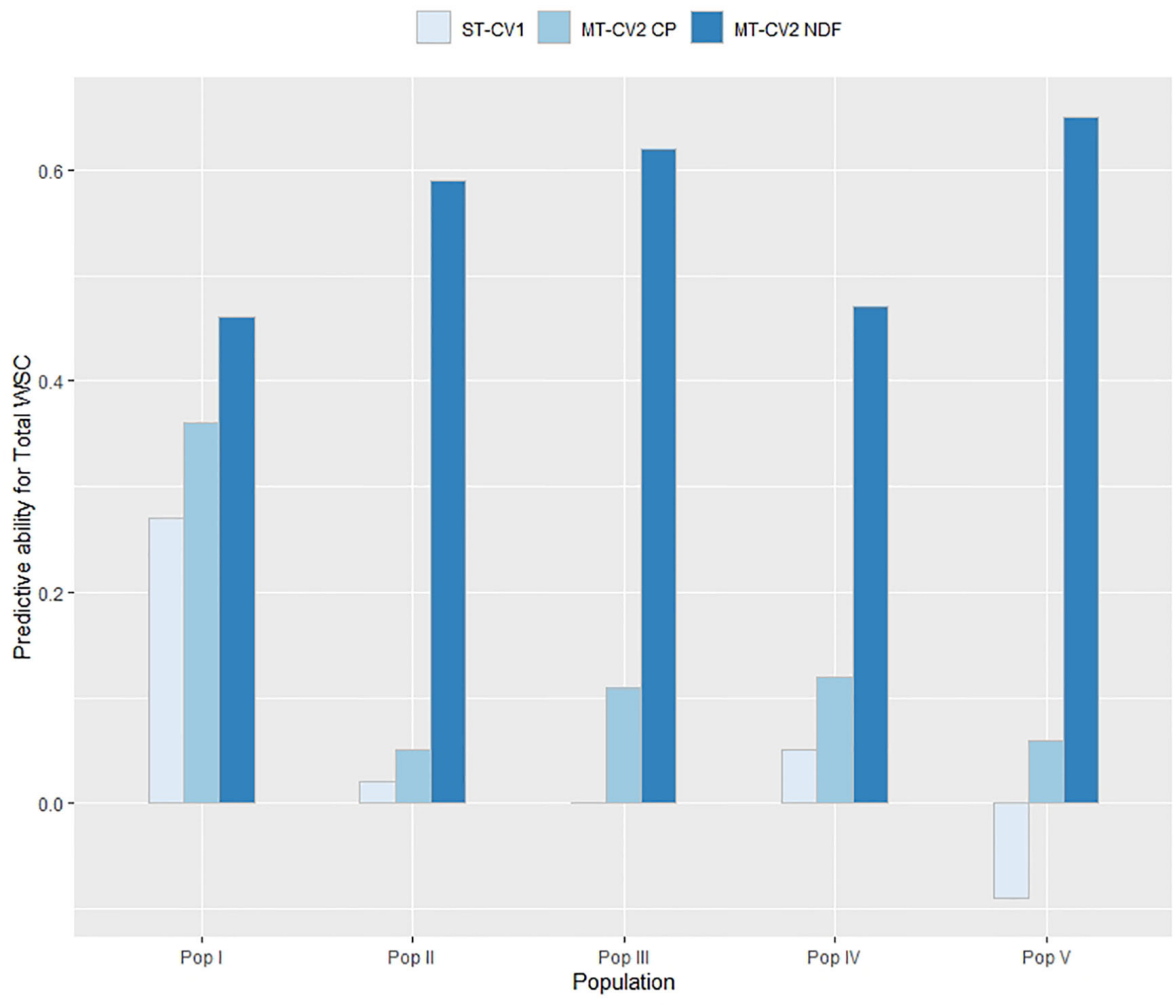
Predictive ability for Total WSC in each population within TP2 was estimated for genomic prediction models using genotypic and phenotypic data from four populations as a training set and the remaining fifth population as a test set. This was repeated five times with a different population as test set each time. Predictive ability was estimated using single trait (ST-CV1) and multi-trait (MT-CV2) genomic prediction models, with the MT-CV2 model further assessed by comparing use of a low correlated secondary trait (CP) and a high correlated secondary trait (NDF).

In TP2, similar results were observed as for TP1 (**Figure 8**). Using the ST-CV1 approach, the genomic prediction model failed to predict Total WSC in Pop III (*r_p_* = 0.0) and Pop V (*r_p_* = −0.09) and the highest predictive ability was seen in Pop I (*r_p_* = 0.27). Using a low correlated secondary trait (CP; *r_g_* = −0.29) in the MT-CV2 model, predictive ability was positive for all five populations but still relatively low (*r_p_* = 0.05 to 0.36). Predictive ability was highest for all five populations when using a highly correlated secondary trait (NDF; *r_g_* = −0.63) in MT-CV2, with values ranging from 0.46 to 0.65 (**Figure 8**).

### Minimum Training Set Size

**Figure 9:**
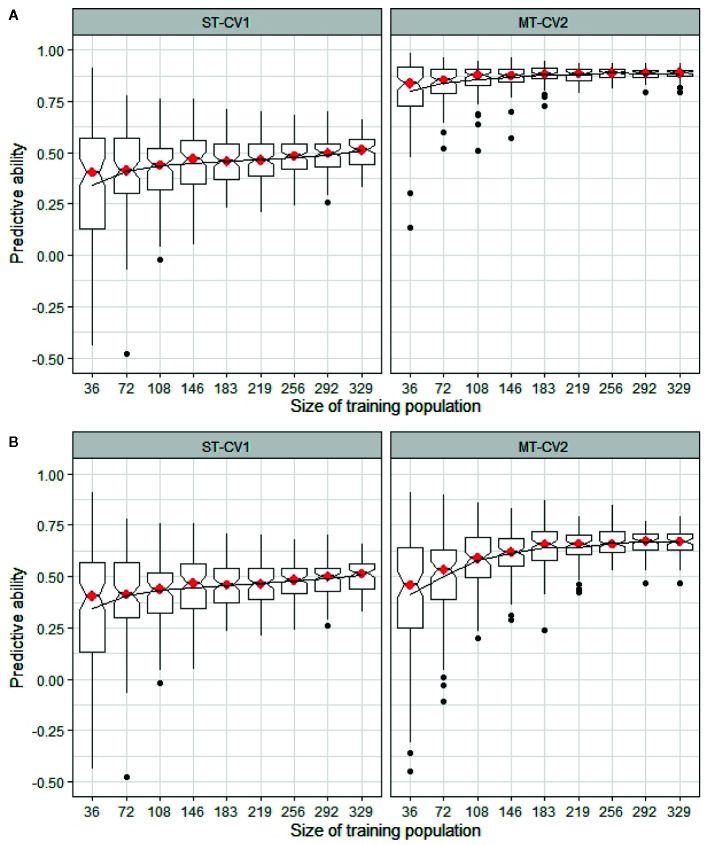
Predictive ability for mean dry matter yield across all seasonal harvests (DMY All Cuts) estimated in TP1 using different training sets, ranging from 36 individuals (10% of the full training set size) to 329 individuals (90%) from training population TP1. Predictive ability was estimated using single trait (ST-CV1) and multi-trait (MT-CV2) genomic prediction models. **(A)** compares predictive ability using ST-CV1 and MT-CV2 models with a highly correlated secondary trait (GS All, *r_g_* = 0.88). **(B)** compares ST-CV1 and MT-CV2 model using a low correlated trait (GS Spring, *r_g_* = 0.56) as a secondary trait.

**Figure 10:**
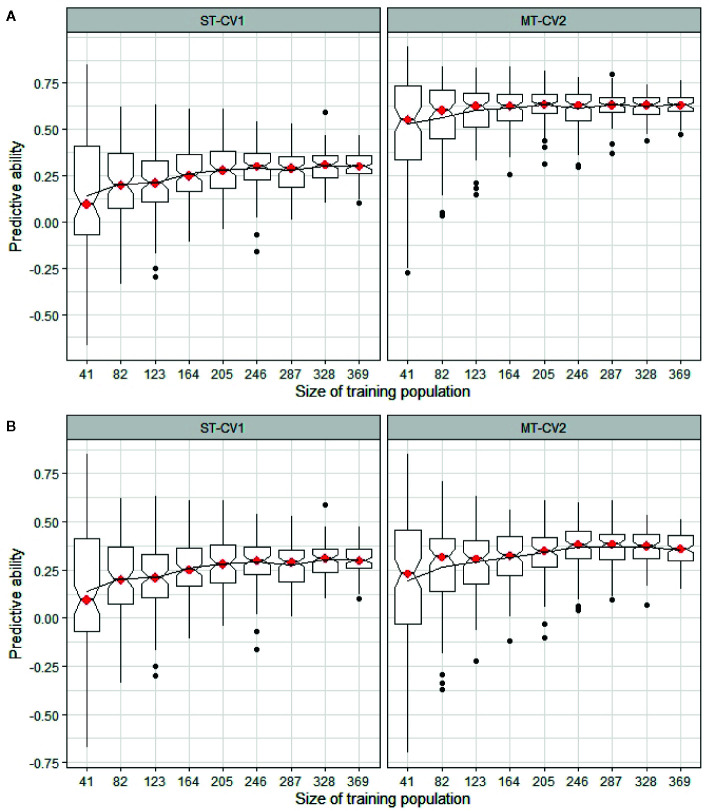
Predictive ability for Total WSC in TP2 was estimated using different training sets, ranging from 41 individuals (10% of full training set size) to 369 individuals (90%) from training population TP2. Predictive ability was estimated using single trait (ST-CV1) and multi-trait (MT-CV2) genomic prediction models. **(A)** compares predictive ability using ST-CV1 and MT-CV2 model with a highly correlated secondary trait (NDF, *r_g_* = −0.63). **(B)** compares ST-CV1 and MT-CV2 model using a low correlated secondary trait (CP, *r_g_* = −0.29).

The minimum training population size was determined in both TP1 and TP2, comparing a highly correlated and a weakly correlated secondary trait in the MT-CV2 model with the predictive ability from a ST-CV1 model (**Figure 9**). In TP1, using trait information for a highly correlated secondary trait (GS All) in both training and test sets, the training set size could be reduced by 50% (n = 183) from its original size, before impacting predictive ability (**Figure 9A**). In the case, where a weakly correlated trait was used as a secondary trait (GS Spring), the training set size was reduced by only 11% from its original size (n = 292) before predictive ability began to decline (**Figure 9B**). In both these situations, the predictive ability was higher in MT-CV2 compared to the ST-CV1 model at the same training set size. Similarly, in TP2, using a highly correlated secondary trait (NDF; *r_g_* = −0.63) for prediction of Total WSC, the point at which predictive ability began to decline was when the training set size was reduced by 22% (n = 287) from its original size (**Figure 10A**). Using a weakly correlated secondary trait (CP and Total WSC; *r_g_* = −0.29), this reduction in predictive ability occurred when the training set had declined by 11% (n = 328) from its original size (**Figure 10B**).

### Optimum GS Secondary Trait for DMY Prediction

In TP1, GS traits were phenotyped in each of 3 years from Spring to Winter, at both locations. The predictive ability of each of these five seasonal secondary traits was assessed in an MT-CV2 model, for prediction of DMY All Cuts (which represents average DMY performance across all seasons) as well as for prediction of each of the seasonal DMY traits. For instance, GS Summer was evaluated as a secondary trait to predict DMY All Cuts, DMY Summer, and DMY Autumn (**Supplementary Figures 5** and **6**). This enabled determination of the best season in which to score the secondary trait to support the most accurate prediction of DMY All Cuts in an MT-CV2 model. Amongst the seasonal GS measures, GS Summer, and GS Autumn had the highest potential to predict DMY All Cuts (predictive ability improved by 51% compared to ST-CV1 for both of these) compared to GS Spring (25%) and GS Late Spring (33%) (**Supplementary Figures 5** and **6**). Whilst using GS All as a secondary trait gave the highest improvement in predicting DMY All Cuts (*r_p_* = 0.78) compared to ST-CV1 (**Supplementary Figure 5**), using the best single seasonal measures, GS Summer or GS Autumn, gave a predictive ability for DMY All Cuts (*r_p_* = 0.74) that was only marginally lower than this (**Supplementary Figures 5** and **6**).

## Discussion

The potential of genomic selection for improving genetic gain in forage plant species has been intensively assessed in recent years (Annicchiarico et al., 2015; Fè et al., 2016; Grinberg et al., 2016; Byrne et al., 2017; Arojju et al., 2018; Faville et al., 2018; Guo et al., 2018; Pembleton et al., 2018; Arojju et al., 2020) but, as yet, multi-trait approaches for genomic selection have not been investigated in these species. Here, we have evaluated and demonstrated the potential of multi-trait genomic selection approaches to improve genomic prediction in perennial ryegrass, a major forage species globally, for the traits dry matter yield (DMY) in one training population (TP1) and water-soluble carbohydrate (WSC) in a different training population (TP2).

In the current study, predictive abilities estimated by cross-validation for DMY using single trait (ST) genomic prediction models (range of *r_p_*= 0.31–0.50) were comparable to other studies of perennial ryegrass (Grinberg et al., 2016; Arojju, 2017; Guo et al., 2018; Pembleton et al., 2018). Predictive ability for DMY reported by Faville et al. (2018) for the TP2 were generally lower than the TP1 values in the current study. The differences in predictive ability for DMY may have been influenced by the phenotypes being collected under different environments and management schemes, as well as contrasting genetic properties of the training sets. Predictive ability for nutritive quality traits using the ST-CV1 model were previously discussed by Arojju et al. (2020). Although prediction models used by Arojju et al. (2020) were different (GBLUP, KGD-GBLUP, and BayesC) to the current study (RKHS), the predictive ability was similar for the majority of traits. Predictive abilities estimated for traits in these training sets could also have been influenced by the genomic selection system adopted, namely the phenotyping of half-sibling families to generate an estimated breeding value for genotyped maternal parents, rather than direct phenotyping the maternal parent itself. In this scenario predictive ability might be affected by genotypic imbalance in the half-sibling row plot used to generate the phenotypic data. Use of family-pool genotyping methods, similar to those described by Byrne et al. (2013) and implemented by Fè et al. (2015) and Guo et al. (2018) may be more reliable in this regard, if applied directly to field plots, but to our knowledge the relative efficacy of the two approaches has yet to be assessed empirically.

### Multi-Trait Approaches to Improve Predictive Ability

In our study, there was no improvement in predictive ability for the primary trait, when a secondary trait was only included in the training set (MT-CV1, **Figure 1**). Studies conducted in wheat, by Sun et al. (2017) reported similar outcomes, when predicting grain yield using secondary traits NDVI and CT. Predictive ability using MT-CV1 in the study mentioned above was equivalent or slightly higher than ST-CV1 approach. Similarly, in sorghum the genomic prediction of biomass using plant height as a correlated secondary trait, found that the MT-CV1 approach was equivalent in terms of predictive ability to ST-CV1 (Fernandes et al., 2018). Using simulations, Jia and Jannink (2012) stated that the MT-CV1 approach can be expected to improve predictive ability only when a correlated secondary trait with high heritability is used to support prediction of a primary trait with low heritability. In our study, in both TP1 and TP2, the genomic heritability estimated for the primary traits were in a similar range as those estimated for the secondary traits (ADF *h^2^_g_* = 0.32 and Total WSC *h^2^_g_* = 0.31) or even higher for some traits (DMY All Cuts *h^2^_g_* = 0.65 and GS All *h^2^_g_* = 0.35) (**Table 1** for TP1; for TP2 refer **Table 1** in Arojju et al. (2020)). Furthermore, the training populations used were relatively small (TP1 = 463 and TP2 = 517). These two factors may have constrained the effectiveness of the MT-CV1 model to improve predictive ability in our study.

In contrast to MT-CV1, the inclusion of a correlated secondary trait in both the training and test set (MT-CV2, **Figure 1**) substantially improved the predictive ability for DMY in TP1 and for WSC traits in TP2, compared to the ST genomic prediction models (**Figures 4** and **5**). This finding is in agreement with previous studies in wheat (Rutkoski et al., 2016; Sun et al., 2017; Crain et al., 2018; Sun et al., 2019) and sorghum (Fernandes et al., 2018), which showed that including secondary traits in both training and test sets can improve genomic predictive ability for the primary trait. In those studies the extent of improvement in predictive ability was dependent upon the heritability and the level of correlation between the primary and secondary traits (Rutkoski et al., 2016; Sun et al., 2017; Fernandes et al., 2018). In TP1, we observed moderate to high correlation between DMY and GS traits. In particular, GS All, GS Summer, and GS Autumn were highly correlated with DMY All Cuts (*r_g_* = 0.80 to 0.88). In TP2, nutritive traits DOMD and NDF were highly correlated with LMW, HMW, and Total WSC traits (*r_g_* = 0.36 to 0.57 for DOMD and *r_g_* = −0.44 to −0.63 for NDF). Inclusion of these highly correlated traits in the MT-CV2 model resulted in the highest improvement in predictive ability. Among nutritive traits, CP was poorly correlated with LMW (*r_g_* = −0.19), HMW (*r_g_* = 0.11) and Total WSC traits (*r_g_* = −0.08). Inclusion of CP as secondary trait in the MT-CV2 model resulted in low or negative improvement in predictive ability. Genomic heritability estimates for the secondary traits were lower than for the primary traits, in both TP1 and TP2. This implies that, in our study, it was the level of correlation between primary and secondary traits, rather than trait heritability, that made a more substantial contribution to improvement in predictive ability for DMY in TP1 and WSC in TP2 when using the MT-CV2 approach.

We also assessed the predictive ability of a model that includes secondary trait information only in the test set (MT-CV3, **Figure 1**) and found an improvement in predictive ability for DMY (TP1) and WSC traits (TP2), compared to the ST genomic prediction models (**Figures 5** and **6**). The MT-CV3 model is therefore another potential approach for improving predictive ability in a genomic selection scheme, but the variation in predictive ability amongst iterations was higher than MT-CV2 (**Figures 5** and **6**). This was also reflected in the slope of regression (bias), which was highly variable compared to the MT-CV2 approach (**Supplementary Figures 3** and **4**). This variability could be due to the limited amount of secondary trait information used in the model (30% in MT-CV3 compared to 100% in MT-CV2 model) (**Figure 1**). In situations with a sufficiently large test set and a high correlation between primary and secondary traits, the MT-CV3 model could be an attractive approach for the prediction of complex traits with higher accuracy. However, when MT genomic prediction models are to be applied at the scale used in the current study, the MT-CV2 approach is likely to produce an equally high and more reliable improvement in genomic predictive ability. A slight increase in bias (bias > 1) was observed among MT-CV2 and MT-CV3 models compared to ST-CV1 model (**Supplementary Figures 3** and **4**). An increase in bias suggests that the predictions were underestimated from the current model (Neves et al., 2012; Neves et al., 2014; Tsuruta et al., 2019). Since the MT predictions models are complex and have recently gained importance in plant and animal breeding, there is no sufficient knowledge to determine the basis for the deflation of bias (bias > 1) specific to MT model. This will be considered as an important issue to be addressed in future studies.

### Optimizing MT-CV2 Multi-Trait Genomic Selection

Application of the MT-CV2 genomic selection approach may enable further efficiencies through the reduction of training population size without compromising predictive ability. Using the MT-CV2 approach, Lado et al. (2018) were able to reduce the training population size to 30% of the original size without an impact on predictive ability for baking quality traits in wheat. We used MT-CV2 results in the current study as the basis for testing the impact of training population size, comparing inclusion of a highly correlated and a low correlated secondary trait in the MT-CV2 model. We found that the training population size could be reduced to 50% of its original size without an impact on predictive ability but only when a highly correlated trait was used as the secondary trait. When using a low correlated secondary trait in the MT-CV2 model the size of the training population size could only be reduced by 11% before predictive ability was negatively affected.

The acquisition of GS measurements across multiple seasons and years is expensive and determining a single, optimum seasonal timepoint to score secondary traits would reduce resource requirements and increase the viability of adopting the MT-CV2 genomic prediction approach for DMY genomic selection. The current dataset indicates that the optimum season for phenotyping visual GS as a secondary trait was during summer and autumn, enabling a predictive ability for DMY close to that of using GS measurements across multiple seasons and years. In TP1 improvement in predictive ability for DMY All Cuts using GS All Cuts as the secondary trait in the MT-CV2 model was 0.78. However, using visual GS measured across multiple years in either summer (GS Summer) or autumn (GS Autumn) alone as the secondary trait, gave a predictive ability of 0.74 (**Figure 5**).

### Genomic Prediction in an Independent Population

The multi-population nature of the training set used in this study enabled investigation of the efficacy of predicting GEBVs in a population unrelated to the training set. When a ST genomic prediction model was trained using data from four populations and GEBVs were predicted for the fifth population, predictive ability was low or negative for most of the populations in both TP1 and TP2 (**Figures 7** and **8**). The reasons for prediction failure could be the lack of genetic relatedness between training and test set, differences in LD between marker and QTL across the different populations, QTL segregating in one population but not be present in another population, and differences in minor allelic frequencies between the populations (Hayes et al., 2009; Zhong et al., 2009; Raymond et al., 2018). We further explored this approach by using either a low or highly correlated secondary trait in a MT-CV2 genomic prediction model and demonstrated that, when applied to an independent population the predictive ability can be positive and high (**Figures 7** and **8**) (**Supplementary Table 5** and **6**). The practical implication of this approach is immense, as it suggests that a MT prediction model developed for complex and difficult to measure traits in breeding program “A” might also be implemented in program “B,” assuming populations in breeding program “B” have been phenotyped for the secondary trait. Initial analysis suggests that the reason for an improvement in predictive ability was due to the correlation between the primary and secondary traits in each population (**Supplementary Tables 5** and **6**). Further investigation is needed to understand the results and its practical implications.

### Genomic Breeding Using Multi-Trait Approaches

For traits such as DMY in forage breeding, which require a high level of effort for multi-environment phenotyping (Conaghan et al., 2008), data acquisition in large training sets (n > 2000) remains a major limitation for implementing genomic selection. This limitation may be overcome by completely phenotyping large training sets for secondary traits (which are relatively inexpensive and can be phenotyped at large scale) and only partially for the primary target trait (difficult and/or expensive to phenotype), followed by applying a MT prediction model to predict the primary trait for selection. Because forage breeding programs often include a single plant evaluation stage (Conaghan and Casler, 2011), a genomic breeding strategy for DMY based on MT-CV2 could be designed to leverage single plant traits that are more easily phenotyped at scale and which have some level of correlation to sward yield, for example leaf length (Rhodes and Mee, 1980; Wilkins and Humphreys, 2003; Barre et al., 2015), plant height (Wang et al., 2016) or, in specific circumstances, single plant vigour (Lazenby and Rogers, 1964; Burton, 1985; Casler et al., 1997). Based on our findings, we expect evaluation of a yield-correlated, single plant trait in a large population sample (e.g. n > 2000) and of the primary trait, DMY, in half-sib families derived from a smaller subset (e.g. n = 400) of that population, could enable enhanced genomic predictive ability for DMY in the full population.

For nutritive quality traits, wet laboratory procedures are generally expensive and time-consuming. Obtaining NIRS data is regarded as a simpler, less expensive alternative but is still sample-destructive and relatively laborious (sample harvest, freeze-drying, milling, and scanning). Recent developments in non-destructive NIR technology could be an option to explore, but to obtain reliable data from non-destructive NIR, multiple scans are needed which adds extra time and cost (Martínez et al., 1998). Recently, hyperspectral imaging (HSI) has attracted interest, due to its ability to capture both spectral and spatial information and its adaptability to work for in-field conditions (Gutiérrez et al., 2018). There has been steady progress in the development of HSI systems for predicting nutritive quality traits in perennial ryegrass (Shorten et al., 2019) and deployment of in-field high-throughput phenotyping system using Light Detection and Ranging (LiDAR) sensor for yield (Ghamkhar et al., 2019). Combining these two technologies and associating spectral reflectance and absorbance with the primary trait of interest (which would be partially phenotyped by wet-lab analysis in the training set) could produce phenotypic information for multiple traits within a single experimental step. Phenotypes derived using HTP sensor systems can be considered as secondary traits, suitable to be rapidly phenotyped across a large training population and used in a MT genomic prediction model to predict the primary trait of interest in selection candidates.

## Conclusion

In this study, we have shown that applying MT genomic prediction can improve predictive ability for DMY or WSC when compared to a ST model. Inclusion of a correlated secondary trait, either in both training and test set (MT-CV2) or in the test set alone (MT-CV3), was highly effectual for enhancing predictive ability. The improvement in predictive ability was in line with the degree of correlation between primary and secondary trait. In contrast, when the secondary trait was included only in the training set (MT-CV1), there was no improvement in predictive ability for either DMY or WSC. The MT-CV2 approach opens up the possibility to assemble larger training sets needed to implement genomic selection for resource-intensive and complex traits in forage species, whereby the full training set is phenotyped for an inexpensive secondary trait and a smaller subset for the primary trait. Use of MT genomic prediction models also appears to be a promising approach for successfully implementing genomic prediction in populations unrelated to the training set, if the independent population has phenotypic data for the secondary trait. MT genomic prediction approaches, coupled with HTP systems, has great potential to accelerate the rate of genetic gain in forage species for key economic traits such as DMY and nutritive quality.

## Data Availability Statement

**Supplementary Material Table 1** consists of phenotypic data for dry matter yield (DMY) and growth scores measured on individuals from training set TP 1 and **Datasheet 2** contains the genomic relationship matrix. **Datasheet 1** contains **Tables 1** to **6** and **Figures 1** to **6** generated in the current study to support the conclusions. The TP 2 genomic relationship matrix and phenotypic data for nutritive quality traits can be available to download at figshare: https://doi.org/10.25387/g3.10074323.

## Author Contributions

MF and BB conceived, designed, and coordinated the study. SA, MC, and MF performed the analysis. MT and CI coordinated the data collection for training population TP2. SA and MF drafted the initial manuscript. SA, MF, MC, BB, MT, CE, CI and AS contributed to interpretation of results and preparation of the final manuscript. All authors contributed to the article and approved the submitted version.

## Funding

This work was supported by Pastoral Genomics (PSTG1501), a research consortium funded by the Ministry of Business, Innovation and Employment (MBIE), DairyNZ, Beef + Lamb New Zealand, Dairy Australia, AgResearch Ltd, Barenbrug Agriseeds Ltd and Grasslands Innovation Ltd.

## Conflict of Interest

Authors SA, MF, BB, MC, and MT are employed by AgResearch, a New Zealand Crown Research Institute. Authors CE and CI are employed by Barenbrug Agriseeds Ltd, and AS is employed by PGG Wrightson Seeds Ltd.
