# Supplementary material for: Multi-Trait Genomic Prediction Improves Predictive Ability for Dry Matter Yield and Water-Soluble Carbohydrates in Perennial Ryegrass
Source: Front Plant Sci. 2020 Aug 7;11:1197. doi: 10.3389/fpls.2020.01197 (PMC7426495; doi:10.3389/fpls.2020.01197)
Supplement: Supplementary file 2 [file DataSheet_1.pdf]

Supplementary Tables:

Supplementary Table 1 Measurement dates for visual growth score (GS) and dry matter yield (DMY) data acquired at two locations (Ruakura and Darfield) and three years for training population TP1. Tick symbol (✓) = data collected and empty cell = no data collected.

Measurement dates	GS - Ruakura	GS - Darfield	DMY - Ruakura	DMY - Darfield
Jul-16	✓			
Aug-16	✓			
Sep-16	✓	✓		
Oct-16		✓	✓	✓
Nov-16	✓	✓		
Dec-16	✓	✓		
Jan-17	✓	✓		
Feb-17	✓	✓	✓	✓
Mar-17	✓			
Apr-17	✓	✓		
May-17	✓	✓	✓	✓
Jun-17	✓			
Jul-17				
Aug-17	✓	✓		
Sep-17	✓		✓	✓
Oct-17	✓	✓		
Nov-17	✓	✓	✓	✓
Dec-17	✓			
Jan-18	✓	✓		
Feb-18	✓		✓	✓
Mar-18	✓			
Apr-18	✓	✓		
May-18	✓	✓	✓	✓
Jun-18				
Jul-18	✓			
Aug-18	✓	✓		
Sep-18	✓		✓	✓
Oct-18		✓		
Nov-18	✓		✓	✓
Dec-18	✓			
Jan-19	✓	✓		
Feb-19	✓		✓	✓
Mar-19				
Apr-19	✓	✓		
May-19	✓			

Supplementary Table 2: Training population TP1 was measured for dry matter yield (DMY) and growth scores (GS) at Ruakura for three years. Shown are family genetic variance (σ^2_g), family-by-measure interactions (σ^2_{gm}) and associated standard error (SE) estimated among perennial ryegrass half-sib families. σ^2_g is significant ($P < 0.05$) for all traits. Data for genomic heritability (h^2_g), predictive ability (r_p) and bias are also provided.

**Trait**	**σ^2_g ± SE**	**σ^2_{gm} ± SE**	**h^2_g**	**r_p**	**bias**
DMY All Cuts	30.69 ± 2.571	3.25 ± 0.637	0.92	0.46	1.01
DMY Spring	19.87 ± 2.836	0.81 ± 0.876	0.63	0.29	0.92
DMY Late Spring	24.95 ± 3.440	0.00 ± 0.000	0.62	0.31	0.98
DMY Summer	24.88 ± 2.770	5.94 ± 1.075	0.77	0.43	1.02
DMY Autumn	14.91 ± 1.975	6.97 ± 0.980	0.66	0.47	1.02
GS All Cuts	0.72 ± 0.053	0.22 ± 0.008	0.96	0.62	1.02
GS Spring	0.35 ± 0.04	0.01 ± 0.012	0.73	0.25	0.89
GS Late Spring	0.27 ± 0.038	0.02 ± 0.020	0.61	0.38	1.04
GS Summer	0.61 ± 0.054	0.19 ± 0.016	0.89	0.58	1.04
GS Autumn	1.07 ± 0.088	0.26 ± 0.017	0.92	0.62	1.03
GS Winter	0.33 ± 0.039	0.23 ± 0.020	0.74	0.36	0.99

Supplementary Table 3: Training population TP1 was measured for dry matter yield (DMY) and growth scores (GS) at Darfield for three years. Shown are family genetic variance (σ^2_g), family-by-measure interactions (σ^2_{gm}) and associated standard error (SE) estimated among perennial ryegrass half-sib families. σ^2_g is significant ($P < 0.05$) for all traits. Data for genomic heritability (h^2_g), predictive ability (r_p) and bias are also provided.

**Trait**	**σ^2_g ± SE**	**σ^2_{gm} ± SE**	**h^2_g**	**r_p**	**bias**
DMY All Cuts	19.97 ± 2.077	7.91 ± 1.051	0.82	0.24	0.85
DMY Spring	5.12 ± 1.073	5.81 ± 1.438	0.31	0.25	0.85
DMY Late Spring	8.50 ± 2.386	7.05 ± 2.424	0.29		
DMY Summer	19.47 ± 3.261	12.86 ± 2.365	0.55	0.40	0.98
DMY Autumn	16.78 ± 3.383	0.00 ± 0.000	0.40	0.27	0.91
GS All Cuts	0.10 ± 0.011	0.12 ± 0.009	0.78	0.30	0.89
GS Spring	0.02 ± 0.007	0.15 ± 0.017	0.16	0.20	0.80
GS Late Spring	0.03 ± 0.017	0.19 ± 0.032	0.09	0.27	0.92
GS Summer	0.13 ± 0.020	0.14 ± 0.016	0.56	0.29	0.90
GS Autumn	0.06 ± 0.013	0.04 ± 0.015	0.38	0.25	0.85
GS Winter	0.11 ± 0.025	0.02 ± 0.022	0.30		

Supplementary Table 4: Training population TP2 was measured for nutritive traits. Each trait was assessed for predictive ability (r_p) at Lincoln and Aorangi and bias of the predictions was estimated.

**Trait**	**Lincoln r_p**	**Lincoln Bias**	**Aorangi r_p**	**Aorangi Bias**
ADF	-0.06	-0.08	0.29	0.91
NDF	0.02	0.30	0.35	0.97
DOMD	-0.02	0.20	0.25	0.94
CP	0.14	0.66	0.21	0.94
LMW WSC	-0.03	-0.34	0.33	0.89
HMW WSC	0.13	0.83	0.56	1.04
Total WSC	0.08	0.52	0.46	0.99

Supplementary Table 5: Pearson correlation coefficient between combined dry matter yield (DMY All Cuts) and growth scores (GS Spring and GS All Cuts) evaluated amongst individual populations (Pop B1 – Pop B5) in training population TP1.

Population	Trait	GS Spring	GS All Cuts
Pop B1	DMY All Cuts	0.69	0.91
Pop B2	DMY All Cuts	0.59	0.83
Pop B3	DMY All Cuts	0.44	0.78
Pop B4	DMY All Cuts	0.50	0.82
Pop B5	DMY All Cuts	0.75	0.95

Supplementary Table 6: Pearson correlation coefficient between Total WSC and CP and NDF evaluated amongst individual populations (Pop I – Pop V) in training population TP2.

Population	Trait	CP	NDF
Pop I	Total WSC	-0.46	-0.45
Pop II	Total WSC	-0.27	-0.64
Pop III	Total WSC	-0.22	-0.68
Pop IV	Total WSC	-0.43	-0.56
Pop V	Total WSC	-0.23	-0.69

Supplementary Figures:

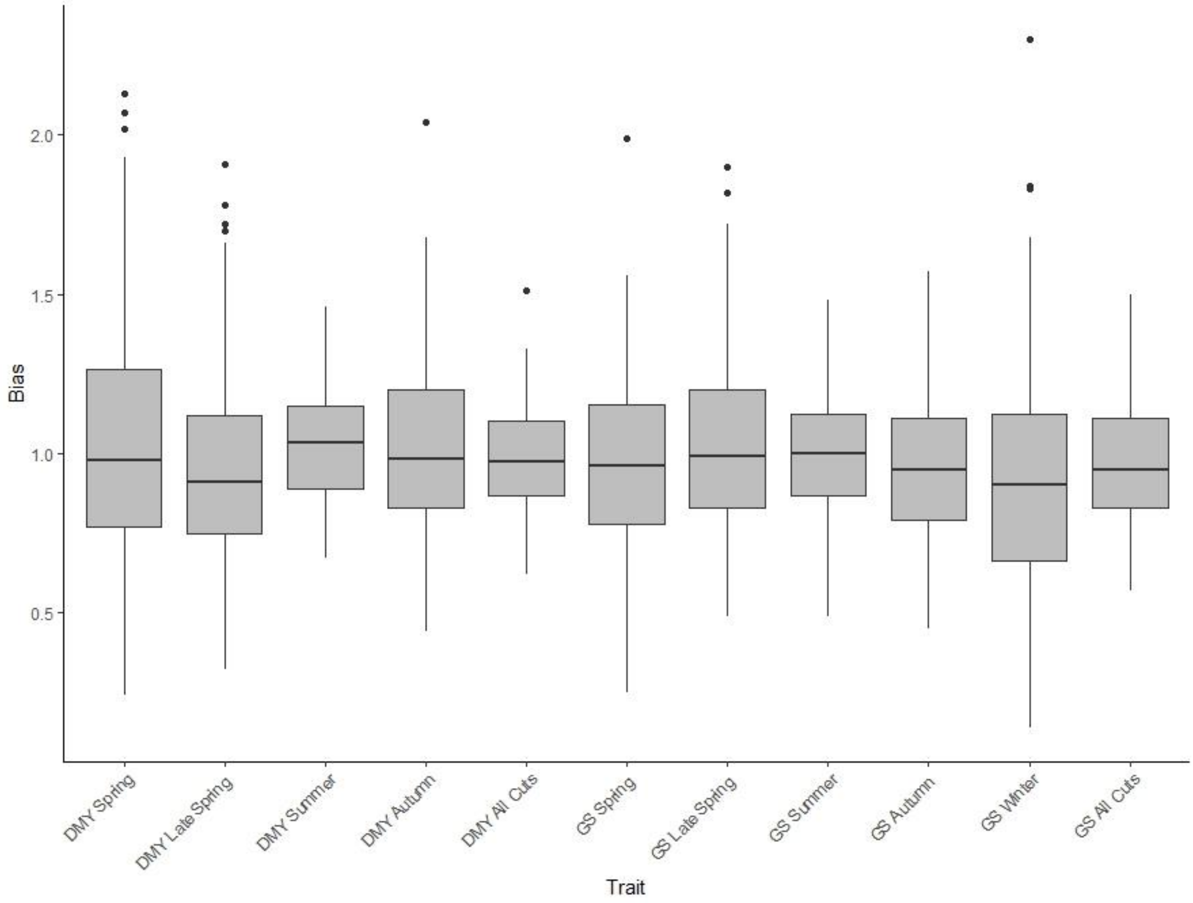

Supplementary Figure 1 Bias for combined (All Cuts) and seasonal dry matter yield (DMY) and growth scores (GS) measured in training population TP1 using the single trait genomic prediction model (ST-CV1 approach) based on across location best linear unbiased predictors (BLUPs).

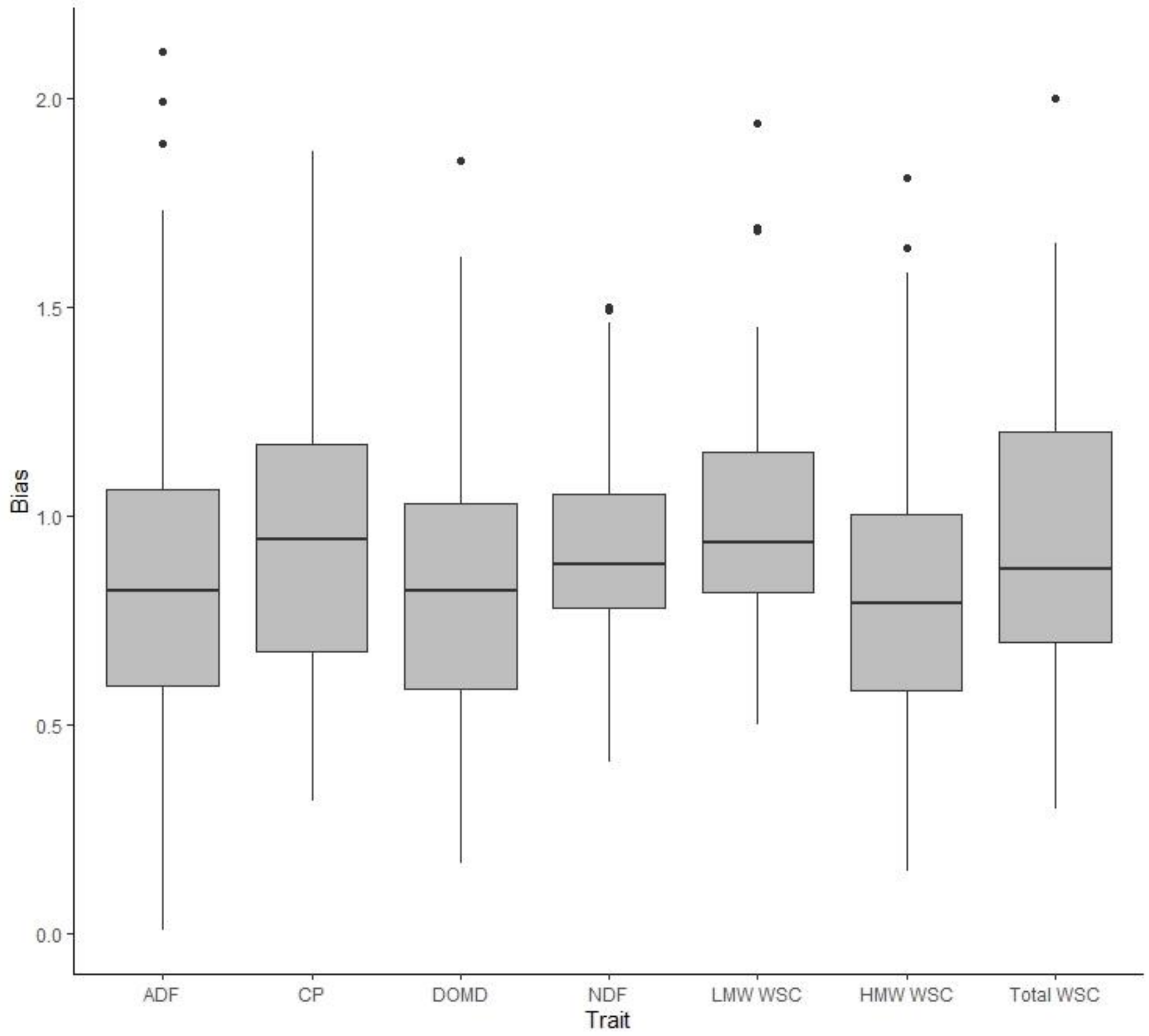

Supplementary Figure 2 Bias for nutritive traits measured in training population TP2 using the single trait genomic prediction model (ST-CV1 approach) based on across location best linear unbiased predictors (BLUPs).

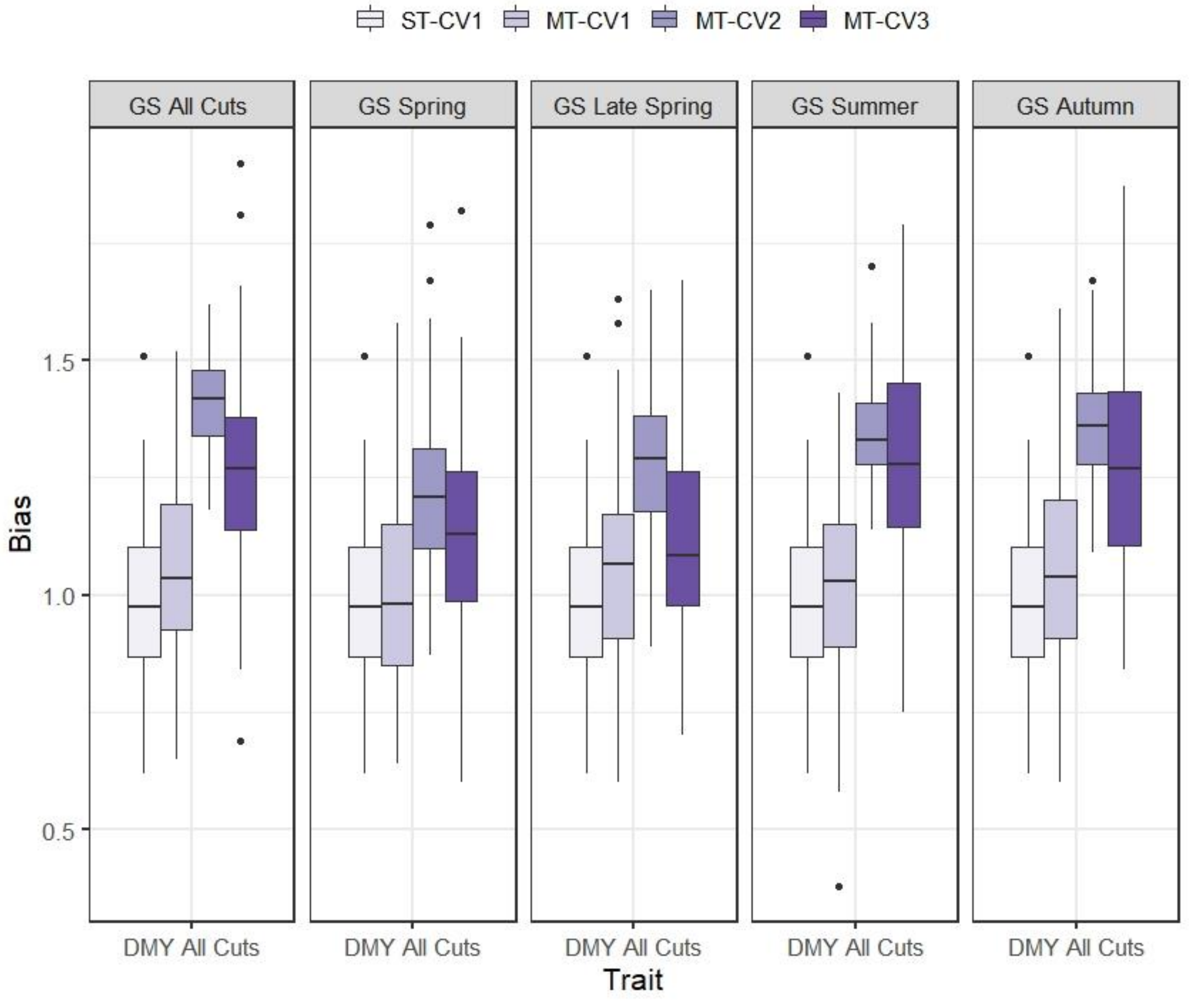

Supplementary Figure 3 Bias for DMY All Cuts measured in training population TP1 using single trait (ST-CV1) and multi-trait (MT-CV1, MT-CV2 and MT-CV3) genomic prediction approaches.

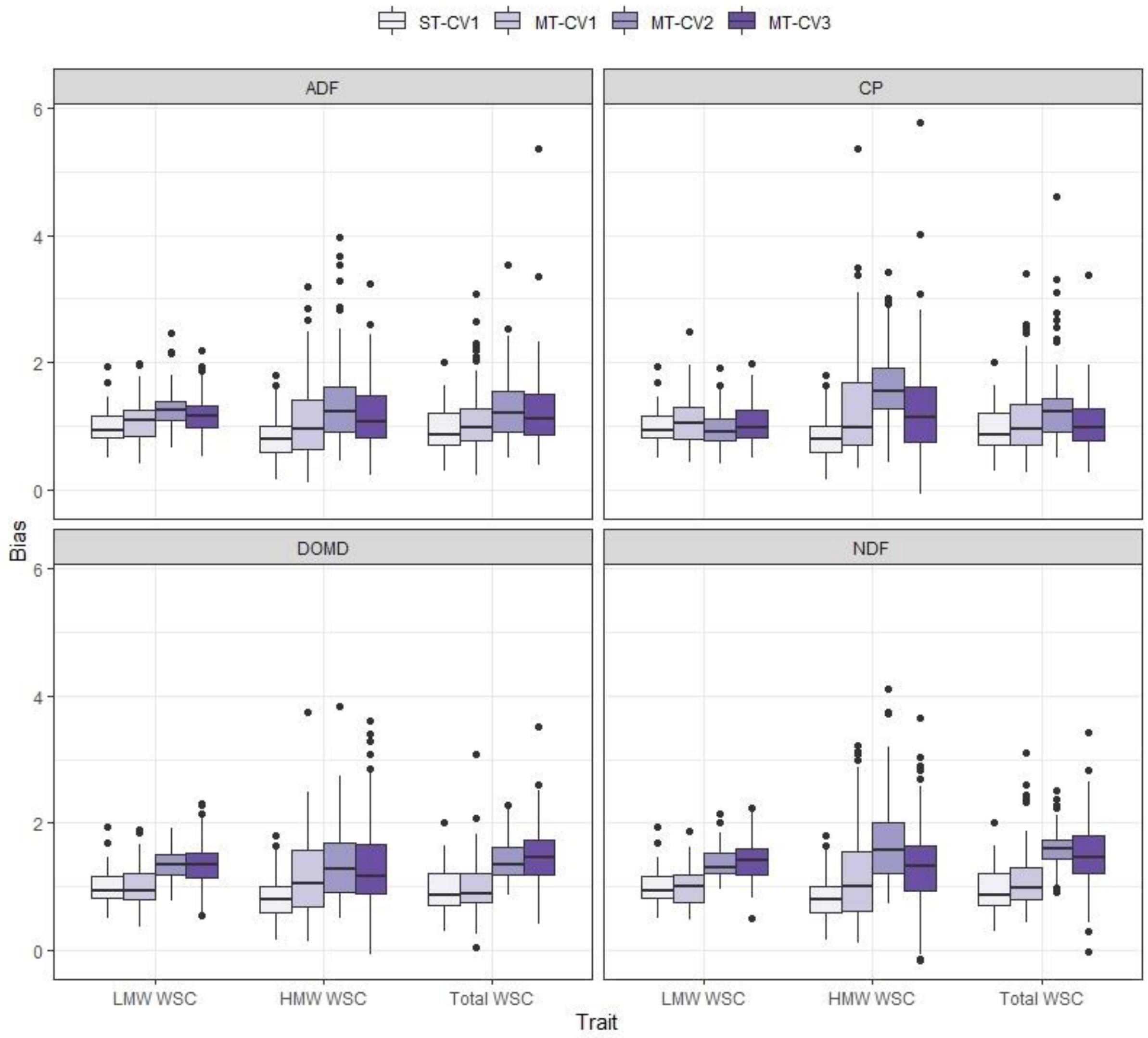

Supplementary Figure 4 Bias for LMW, HMW and Total WSC traits measured in training population TP2 using single trait (ST-CV1) and multi-trait (MT-CV1, MT-CV2 and MT-CV3) genomic prediction approaches.

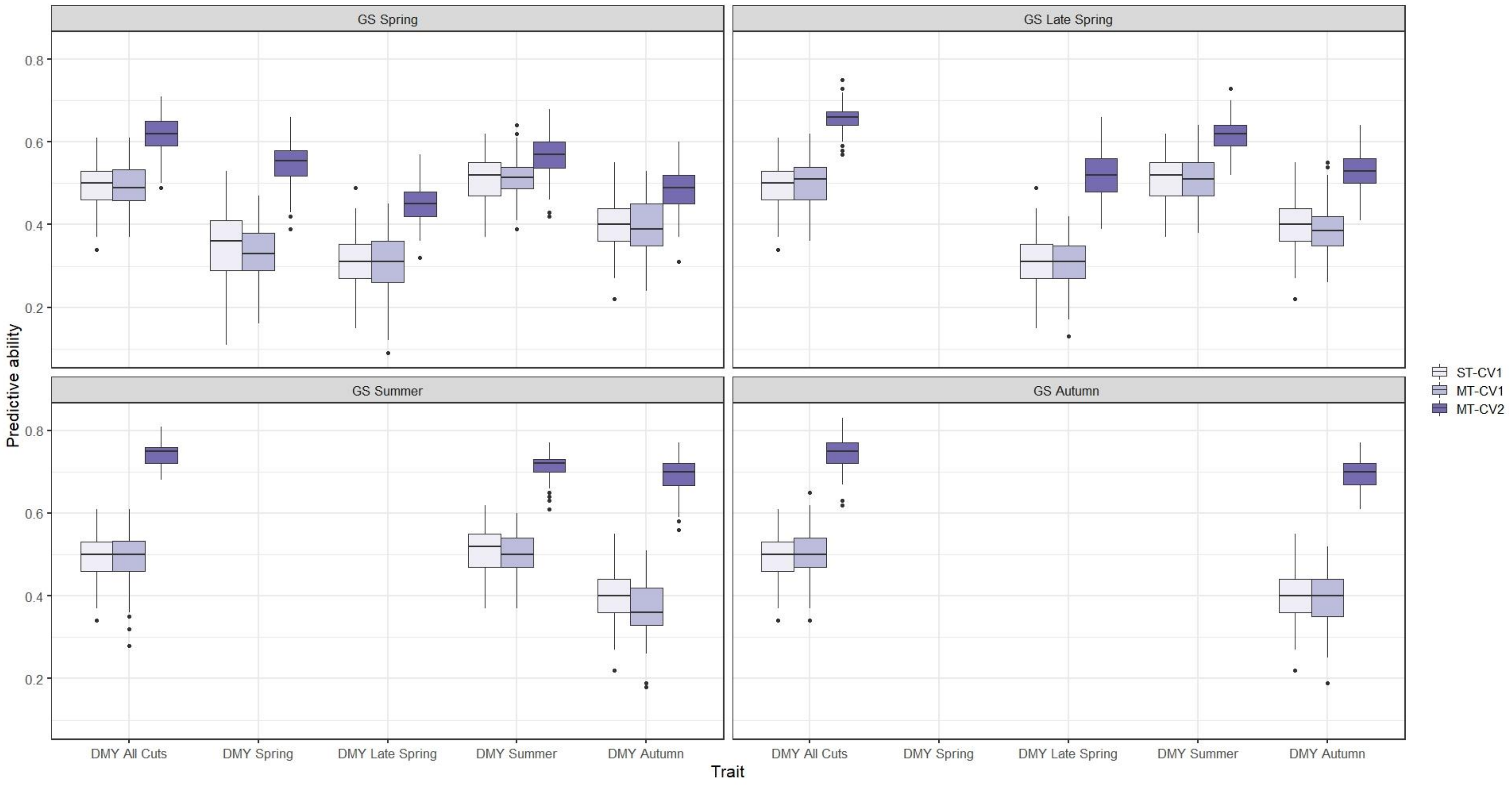

Supplementary Figure 5: Predictive ability for mean dry matter yield in TP1 from all seasonal harvests (DMY All Cuts) and for individual seasonal DMY harvests (Spring, Late spring, Summer, Autumn) using single-trait (ST-CV1) and multi-trait (MT-CV1 and MT-CV2) genomic prediction models. MT genomic prediction models included mean growth scores (GS) measured at each seasonal period as secondary traits.

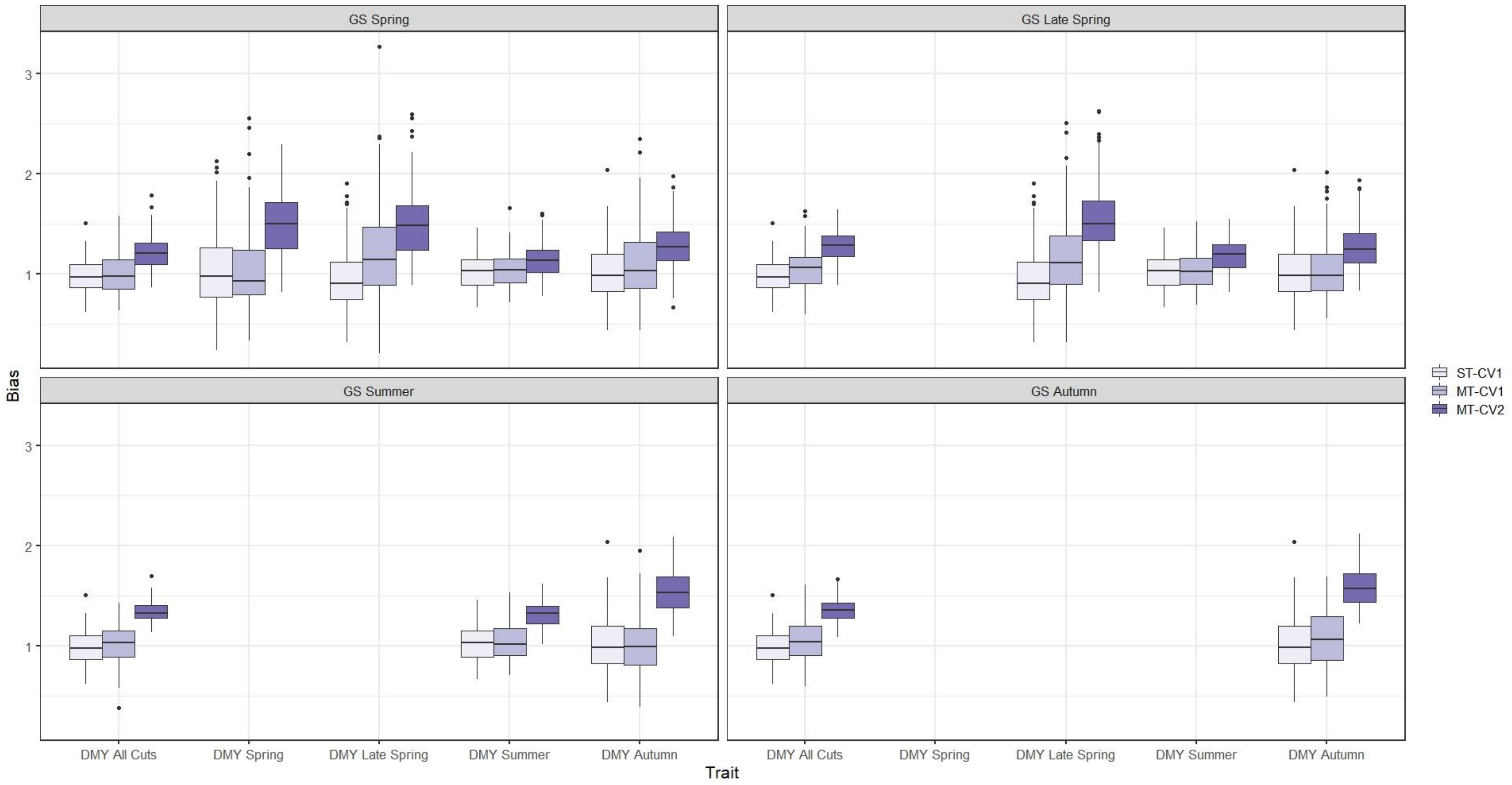

Supplementary Figure 6: Bias for mean dry matter yield in TP1 from all seasonal harvests (DMY All Cuts) and for individual seasonal DMY harvests (Spring, Late spring, Summer, Autumn) using single-trait (ST-CV1) and multi-trait (MT-CV1 and MT-CV2) genomic prediction models. MT genomic prediction models included mean growth scores (GS) measured at each seasonal period as secondary traits.
